# Developmental Relations Between Bullying Victimization and Suicidal Ideation in Middle Adolescence and Emerging Adulthood: Do Internalizing Problems and Substance Use Mediate Their Links?

**DOI:** 10.1007/s10964-022-01630-4

**Published:** 2022-05-14

**Authors:** Xinxin Zhu, Helen Griffiths, Manuel Eisner, Urs Hepp, Denis Ribeaud, Aja Louise Murray

**Affiliations:** 1 Department of Psychology, University of Edinburgh, Edinburgh, UK; 2 Department of Clinical and Health Psychology, University of Edinburgh, Edinburgh, UK; 3 Jacobs Center for Productive Youth Development, University of Zurich, Zurich, Switzerland; 4 Institute of Criminology, University of Cambridge, Cambridge, UK; 5 Integrated Psychiatric Services Winterthur-Zurcher Unterland, Winterthur, Switzerland

**Keywords:** Suicidal ideation, Bullying victimization, Substance use, Internalizing problems, Mediation mechanisms

## Abstract

Previous research has suggested that bullying victimization is associated with higher suicidal risk among young people; however, the mechanisms underlying this relation have not been well examined. The current study aimed to illuminate the developmental links between bullying victimization and suicidal ideation by examining the mediating roles of depressive symptoms, anxiety symptoms, and substance use. The study sample consisted of *n* = 1465 participants (51.7% male) from the normative z-proso study. Using random intercept cross-lagged panel models and three waves of longitudinal data (ages 15, 17, and 20), the hypothesized mediation effects at the within-person level were tested while partialling out between-person confounds. The results suggested that, at the within-person level, bullying victimization did not predict subsequent suicidal ideation via depressive symptoms, anxiety symptoms, or substance use. However, age 15 bullying victimization predicted within-person increases in age 17 depressive symptoms and suicidal ideation. In addition, depressive symptoms at age 15 and tobacco and cannabis use at age 17 were associated with within-person increases in bullying victimization at ages 17 and 20, respectively. The results also indicated that cannabis use and suicidal ideation were positively and reciprocally related over time. Future studies collecting data at multiple timescales are needed to understand proximal and longer-term mechanisms underlying the relation between bullying victimization and suicidality.

## Introduction

The elevated risk of suicide among youth exposed to bullying (or peer) victimization is a significant concern (see van Geel et al., 2021, for a meta-analysis), the more so because both bullying victimization (Modecki et al., 2014) and suicidality (incorporating a range of suicidal ideation and behaviors; Cha et al., 2018) are prevalent among young people. A better understanding of the mechanisms that link bullying victimization to suicidality in young people is needed to inform health policies, preventive screening, and interventions (Hong et al., 2015). Thus, the current study aimed to investigate potential mediators of this link, focusing on internalizing problems (i.e., depressive and anxiety symptoms) and substance use (i.e., tobacco, alcohol, and cannabis use). This study focuses on suicidal ideation as the outcome, owing to its strong links to suicidal attempts and completion (e.g., Castellví et al., 2017). To provide a developmental perspective and to help disentangle the processes occurring within young people over time from between-person confounds, this study used the random-intercept cross-lagged panel model (RI-CLPM) in a high-quality longitudinal dataset spanning ages 15 to 20.

### The Potential Mediating Roles of Internalizing Problems and Substance Use

Bullying is commonly defined as repeated intentional aggressive behavior that involves a power imbalance between perpetrators and victims (Olweus, 1993). There is ample evidence suggesting that bullying victimization is associated with subsequent suicidal ideation among youth (see van Geel et al., 2021, for a meta-analysis). However, exposure to bullying victimization may act via triggering certain psychological or behavioral processes that can lead to suicidality, and those processes are not well understood or rigorously examined (Hong et al., 2015).

According to the general strain theory (e.g., Agnew, 2001), for instance, internalization or substance use may function as (maladaptive) coping mechanisms evoked by strains (e.g., bullying victimization). In this view, suicide is conceptualized as a kind of self-directed, escapist coping response (e.g., Walls et al., 2007), which may be a last resort, especially in the absence of effective coping resources to buffer the influence of repeated exposure to uncontrollable interpersonal stressful life events. Moreover, from the three-step theory (3ST, and psychache theory) perspective (Klonsky & May, 2015), suicidal ideation can begin with psychological or emotional pain (e.g., internalizing problems), and bullying victimization/social rejection may be a significant source of eliciting distressing feelings in youth. Combining these two theoretical perspectives (i.e., the general strain and three-step theory), internalizing problems may act as mediators linking victimization experiences and suicidal ideation.

In addition to the aforementioned general strain theory, the effects of substance use on suicidality have been extensively investigated, and the potential mediating role of substance use in the victimization-suicidality link has been tentatively indicated by empirical studies. Possible explanations for the role of substance use as a mediator in the victimization-suicidality link, for example, include the idea that substance use, as one possible coping mechanism for victimization experiences, may impair judgment, worsen impulse control, or increase the risk of neurocognitive dysfunctions, resulting in suicide risk (e.g., Pompili et al., 2012). A related idea is that substance use may be co-occurring with psychopathology (e.g., depressive symptoms, Hunt et al., 2020), which may in turn increase suicide risk. However, a lack of theories incorporates problematic substance use into the association between interpersonal stress and suicidality. Given indications from empirical evidence suggestive of its role, it will be critical to further evaluate it as a mediator to inform the enrichment of suicide theories.

Some empirical studies have investigated mediating effects of depressive and anxiety symptoms, and substance use on the relation between victimization and suicidal ideation. However, the majority of these studies employed a cross-sectional design, making it difficult to discern temporal order. Indeed, previous authors have highlighted that cross-sectional mediation analyses can produce misleading findings (Maxwell & Cole, 2007). For example, a cross-sectional study surveyed 403 (ages 13–16 years) American adolescents and found that traditional/cyber victimization was indirectly related to suicidal ideation through depressive symptoms (Fredrick & Demaray, 2018). Another cross-sectional study, recruiting 12,354 Chinese adolescents (ages 10–21 years), indicated that the effects of bullying victimization, witnessing, and perpetration on suicidal ideation were linked via depressive symptoms and negative coping styles (Duan et al., 2020). Regarding anxiety symptoms, a study based on 488 American adolescents (ages 10–18 years) found no evidence that the indirect effect of anxiety symptoms on the cross-sectional relation between bullying victimization and suicidal ideation when family conflict, depressive symptoms, and substance use were also included in the model (Lardier et al., 2016). However, a longitudinal study showed that bullying victimization predicted self-harm thoughts via emotional problems (reflective of anxiety symptoms) and depressive symptoms in a small group of adolescents (*n* = 112; *M*_*age*_ = 14.14, *SD* = 1.81) from high-risk communities (i.e., respondents with discipline issues) who participated in two interviews at a 6-month interval (Bryson et al., 2020).

Concerning the empirical evidence that on the mediating role of substance use, one cross-sectional study suggested that physical/cyber bullying victimization was associated with substance use (including seven forms of substance use, e.g., alcohol, tobacco) and violent behavior, which was in turn related to suicidality among American adolescents (*n* = 4693, ages 14–19 years; Litwiller & Brausch, 2013). A similar cross-sectional study has also been carried out with American youth (*n* = 15,364, ages 12–18 years; Moon et al., 2015), supporting the indirect effect of substance use (including legal and illegal substance use, e.g., smoking marijuana) on the victimization-suicidality association. A study of 238 Canadian adolescents investigated the longitudinal relations between peer victimization, alcohol use, and suicidal ideation, using a cross-lagged panel model (CLPM) (Marschall-Lévesque et al., 2017). The results suggested that age 13 victimization was associated with age 14 suicidal ideation, which in turn predicted age 15 alcohol use; however, a direct test of mediation was not performed.

### The Importance of Within-Person Associations

The aforementioned studies have made important contributions to the understanding of mediation mechanisms underlying the relation between bullying victimization and suicidal ideation and provide preliminary support for mediating effects of depressive symptoms, anxiety symptoms, and substance use. However, the causal processes assumed by developmental theories (including the current question) and targeted by interventions are typically implicitly or explicitly conceptualized at the within-person level, e.g., the presumption is that increased victimization leads an individual to escalate their substance use, which in turn leads changes in their suicidal ideation (Berry & Willoughby, 2017). This requires techniques that disaggregate within-level and between-level associations in longitudinal data. The between-person level here refers to the idea that frequently victimized youth may use more substances and show greater internalizing problems and suicidal ideation than those who are less or not victimized. This level arguably does not provide useful targets for interventions typically employed in practice over and above that which can be uncovered by within-person analyses since it reflects factors that vary between people but that tend to be stable over time. The mismatch between employing between-person analyses or analyses confounded by the between-person variance to draw conclusions about within-person processes, may lead to a biased conclusion and ineffective/inaccurate intervention guidance, especially as growing evidence indicates that the between- and within-person relation can be different and even can be opposite in sign or direction (e.g., Dietvorst et al., 2018).

### Bidirectional Associations between Bullying Victimization, Substance Use, Internalizing Problems, and Suicidality

A further issue that needs to be addressed is that research evaluating these mediating mechanisms has been mostly limited to the unidirectional pathway from bullying victimization to suicidal ideation, which may oversimplify the complex processes underlying these links (e.g., Zhu et al., 2021). For example, youth suffering from suicidality (e.g., Speckens & Hawton, 2005), depressive symptoms (e.g., Rudolph et al., 2008), anxiety symptoms (e.g., de Lijster et al., 2018), and/or substance use (e.g., Unger et al., 2003) are more likely to display significant social-behavioral deficits such as using less effective, mood-based, or conflict solutions on social problem solving, or engaging more impulsive (e.g., Verdejo-García et al., 2008) and aggressive behaviors (e.g., Doran et al. 2012). As a result, these youth may create negative social circumstances in which they exhibit a distinctive pattern of social behavior that may elicit disliking or aggressive responses from their peers and/or may result in them being perceived as an easy or desirable target, making them more vulnerable to victimization. These speculations on how suicidal ideation, internalizing problems, and substance use can also lead to increases in the risk of bullying victimization are supported by some empirical evidence where they have been examined (see Christina et al. 2021, for a meta-analysis); however, there also remains a need to examine these links using statistical methods that can disaggregate between- and within-person associations.

### Differential Effects of Substance Use Types

Previous evidence has suggested relations between substance use and suicidal ideation might be complex and are not yet fully understood. Specifically, there might be variation in the magnitude and consistency of the associations between different types of substance use with suicide ideation, among which alcohol, tobacco, and cannabis use have received extensive attention. For example, a meta-analysis study indicated positive longitudinal associations between cannabis use in adolescence and suicidal ideation in young adulthood (Gobbi et al. 2019), which is consistent with previous review work (e.g., Schmidt et al. 2020). However, a recent meta-analysis of prospective cohort studies covering a wide range of age groups suggested a significant prospective effect of alcohol use on suicidal attempts and completed suicide but not on suicidal ideation (Amiri and Behnezhad 2020), which was corroborated by another meta-analysis indicating no significant effect of alcohol reduction on suicidal ideation (Witt et al. 2021). This could be because despite the fact that other evidence has shown suicidal ideation to strongly predict suicidal attempts, alcohol use may play a particularly important role in impulsive or unplanned suicidal behavior (e.g., suicide attempt with no prior suicidal ideation) (e.g., Schilling et al. 2009). A few longitudinal studies have focused on the relation between tobacco use and suicidal ideation in young people but yielded mixed findings: Some supported the positive link between the two (e.g., Bronisch et al. 2008) while others did not (e.g., Boden et al. 2008). Although more research is needed, a small number of studies suggested the reverse pathway from suicidal ideation to substance use in young people (e.g., Marschall-Lévesque et al. 2017), which supports the self-medication hypothesis (i.e., substances might be used to cope with or self-medicate suicidal thoughts). Taken together, there is a clear need for further research to clarify the direction of longitudinal relations between suicidal ideation and the use of different substances among young people.

### Increased Suicide Risk in the Transition from Middle Adolescence to Adulthood

The developmental stages from middle adolescence to emerging adulthood (i.e., from ages 15 to 20) were the particular focus of this study, as the suicide rate is higher in older adolescents than in younger adolescents (Glenn et al. 2020). The lifetime prevalence rate of suicidal ideation has been found to be in the range of 19.8–24.0% and 19.5–25.3% for adolescence (see Nock et al. 2008, for a review) and young adulthood (see Mortier et al. 2018, for a review), respectively. Further, the suicide-related risk factors examined in this study, e.g., internalizing problems and substance use, increase as young people age across adolescence and into young adulthood. For example, it is estimated that globally, 34% of youth aged 10–19 years are at risk of developing depressive symptoms (Shorey and Wong 2021), with youth aged 15–19 showing a higher rate of depressive disorder compared to those aged 10–14 (World Health Organization 2021). Concerning substance use, globally, 13.6% of youth aged 15–19 years have engaged in heavy episodic drinking in 2016, and about 4.7% of adolescents aged 15–16 years used cannabis at least once in 2018 (World Health Organization 2021). These have a major individual and societal cost, with mental health issues (World Health Organization 2021) and substance use (Degenhardt et al. 2016) each accounting for more than 10% of the worldwide burden of disease and injury among youth. Though not as common as in early adolescence, bullying victimization remains common at this age), with an average prevalence of 35% for traditional victimization and 15% for cyber victimization among youth aged 12–18 (Modecki et al. 2014). Taken together, the transition from middle adolescence into adulthood may represent a critical period to capture how earlier victimization experiences cascade into later suicide risk.

## Current Study

Extensive literature has suggested that bullying victimization is related to subsequent suicidal ideation in youth and theoretical and empirical evidence suggests that internalizing problems and substance use may mediate this association. However previous studies were limited by cross-sectional design, providing results with mixed within-and between-person variances, as well as mostly failing to consider bidirectional associations. The current study, thus, aimed to examine the bidirectional among these variables and mediating effects of internalizing problems (i.e., depressive and anxiety symptoms) and substance use (i.e., tobacco, alcohol, and cannabis use) on the association between bullying victimization and suicidal ideation at the within-person level using a random intercepts cross-lagged panel model fit to longitudinal data. Based on the above-reviewed theoretical and empirical evidence, this study hypothesized that within-person increases in bullying victimization would predict increases in depressive symptoms, anxiety symptoms, and substance use and that these would, in turn, predict increases in suicidal ideation and mediate the relation between bullying victimization and suicidal ideation. Besides the aforementioned hypothesized pathways, this study built a full model that allows all constructs included in the model to be bi-directionally associated with each and tested the reverse mediating pathway from suicidal ideation to bullying victimization via depressive and/or anxiety symptoms, and/or substance use (for an exploratory purpose). This study also hypothesized, based on previous evidence, that suicidal ideation, internalizing problems, and substance use would predict within-person increases in bullying victimization.

## Methods

### Participants

Data were obtained from the Zurich project on social development from childhood to adulthood (z-proso, https://www.jacobscenter.uzh.ch/en/research/zproso/aboutus.html): An ongoing longitudinal study of Swiss youth with a particular focus on crime and aggression with an initial target sample of N = 1,675. The first data collection was carried out in 2004/5 at age 7 and comprised all Grade 1 classes of 56 primary schools in the city of Zurich selected by a stratified random sampling procedure taking into account school sizes and the socioeconomic background of the school districts. This study used data from the sixth (age 15), seventh (age 17), and eighth (age 20) wave because suicidal ideation was first collected at age 15. As previously mentioned, youth have a heightened suicide risk during this period than in early adolescence. The suicide rate among Swiss youth aged 15–19 was 7.04 per 100,000 (Glenn et al. 2020), and the prevalence rates of depressive and anxiety disorders (with diagnosed) are 3.8% and 10.2% for Swiss youth aged 15–19, respectively (Institute of health Metrics and Evaluation 2019). Moreover, substance use in this period is common among Swiss youth. For example, more than 30% and 50% of Swiss youth (based on the current sample) reported past-year cannabis use at ages 15 and 17 (Shanahan et al. 2021)/age 20 (Quednow et al. 2021), respectively. The rates of the issues listed above are higher in older adolescence (≥age 15) than in younger adolescence (ages 10–14). As such, mental health (including suicide risk) and behavioral issues are more likely to occur during old adolescence, which warrants attention.

Participants with valid data on variables of interest in at least one wave were included in the current analytic sample. A total of 1465 youth (51.7% male) were included in analyses, of whom 1363 participated in the assessments at least twice and 1102 participated in the assessments three times (see Table S1 of supplementary materials, for details). The results showed that participants with complete data (*n* = 1102) showed significantly higher levels of bullying victimization at age 15 (complete vs. incomplete: *Mean* = 1.67, *SD* = 0.70 vs. *Mean* = 1.54, *SD* = 0.62), as well as higher socioeconomic status (the mean International Socioeconomic Index, complete vs. incomplete: *Mean* = 46.63, *SD* = 17.90 vs. *Mean* = 38.12, *SD* = 16.08), compared to those who provided incomplete data (*n* = 363). At ages 15, 17, and 20, 1%, 11%, and 19% (these percentages vary very slightly according to each variable) of the 1465 participants included in the current analysis had missing data, respectively. More detailed information on the z-proso study (e.g., Ribeaud et al., 2021) and comprehensive analyses of its non-response and attrition (e.g., Eisner et al., 2019) were discussed in previous publications.

Basically, school in Switzerland is compulsory up to grade 9 (i.e., age 15/16; corresponds to wave 6 in z-proso). After that, most youth (>90%) continue with some post-compulsory education, with about 1/3 at the baccalaureate level (up to age 18/19, grade 12), which leads to universities, and about 2/3 in vocational training (which lasts for 2–4 years, equivalent to grades 11–13), which is typically a mix of school-based training in vocational schools and apprenticeship in a company. At age 20 (wave 8 in z-proso), many youth (>50%) are still involved in the education process. Accordingly, at ages 15 and 17, participants were asked to complete school-based paper-and-pencil surveys during their leisure time and were compensated accordingly (50/60 Swiss Francs [1 Swiss Franc = approximately 1 USD]). At age 20, they were invited to complete lab-based CAPI surveys during their leisure time and were compensated with 75 Swiss Francs. The survey lasted approximately 60–90 min. All data in this study were self-reported by participants who were guided by trained staff in completing the questionnaire. The z-proso study received approval from the responsible ethics committee at the Faculty of Arts and Social Sciences, University of Zurich, and participants provided active informed consent at each wave included in the current study.

Previous studies, using data from the z-proso study, have also examined bullying victimization in relation to its outcomes, such as internalizing problems (Averdijk et al., 2016), suicidal ideation and self-injury (Zhu et al., 2021), substance use (Obsuth et al., 2018), and violent ideations (Eisner et al., 2021). However, none have applied a within-person approach to examine the mediating mechanisms underlying the victimization-suicidality link.

### Measures

#### Suicidal ideation

Suicidal ideation was measured using an item that asks youth how often they had thought about suicide during the *past month*, and they rated on a five-point scale (1 = *never* to 5 = *very often*). Using this item in the current sample, previous research has provided preliminary support for its validity (e.g., Steinhoff et al., 2020).

#### Bullying victimization

The Zurich Brief Bullying Scales (ZBBS; Murray, Eisner et al., 2021) was used to assess bullying victimization occurring online or offline. The ZBBS was an adaption of a commonly used measure of bullying developed by Olweus (1996), and captures the defining features of “bullying” because it introduced this measure to participants using an equivalent term to “bullying” (i.e., “plagen” in German, which implies a power imbalance) (see Murray, Eisner et al., 2021 for details). The evidence of its factorial validity, reliability, and convergent and divergent validity has been established previously (Murray, Eisner et al., 2021). Based on the previous validation results using the current sample (Murray, Eisner et al., 2021), four items (physical, verbal, social, and property damage) of ZBBS were included in the bullying victimization assessment. The frequency of each experience over the *past year* was assessed, with a six-point scale: 1 = *never*, 2 = *1 to 2-times*, 3 = *3 to 10-times*, 4 = *about once a month*, 5 = *about once a week*, and 6 = *(almost) every day*. Responses were averaged to a composite score, with a high score indicating a high frequency of victimization. In the current analyses, omega coefficients for the four-item victimization scale were .74 (age 15), .77 (age 17), and .72 (age 20).

#### Depressive and anxiety symptoms

Depressive and anxiety symptoms in the *past month* were both measured with the self-reported Social Behavior Questionnaire (SBQ, Tremblay et al., 1991). The two four-item subscales of SBQ assess depressive symptoms (e.g., *I was sad without knowing why*) and anxiety symptoms (e.g., *I was worried*) on a five-point scale ranging from *never* (1) to *very often* (5). Depressive and anxiety symptoms scores were calculated by averaging the scores for the relevant items, with higher scores indicating high levels of depressive or anxiety symptoms. Previous studies have demonstrated the good psychometric properties of the German version of the SBQ in the current sample (e.g., Murray et al., 2017). In the current study, omega coefficients for depressive symptoms subscale were .76 (age 15), .78 (age 17), and .79 (age 20), and for anxiety symptoms subscale were .73 (age 15), .76 (age 17), and .79 (age 20).

#### Substance use

Substance use was measured by providing a checklist of substances with the instruction: “Listed below are some drugs, intoxicants and other substances. Have you ever taken any of them and if yes, how many times in the *last 12 months*)?”. Tobacco, alcohol (beer/alcopops), alcohol (spirits), and cannabis use were included in this study. Each substance was assessed with one item, with a six-point scale: 1 = *never*, 2 = *once*, 3 = *2 to 5 times*, 4 = *6 to 12 times (monthly)*, 5 = *13 to 52 times (weekly)*, and 6 = *53 to 365 times (daily)*. Additional details on assessing substance use can be found in previous publications using the same dataset (Murray, Eisner, Obsuth, et al., 2017).

In the z-proso study, the “past month” timeframe was employed for both suicidal ideation and internalizing problems, while the “past year” timeframe was used for both bullying victimization and substance use. A previous study using the current sample provides a more comprehensive discussion of the rationale for and implications of employing different timeframes and how they can also be used to test bidirectional, within-person relations between victimization and suicidal ideation (see Appendix materials in Zhu et al., 2021). In brief, the rationale for employing different timeframes is related to the original goal of the z-proso temporal design to be able to provide a plausible time order for risk factors (e.g., victimization) and outcomes (e.g., suicidal ideation) even when data from the same wave were used. Although these constructs were measured with different reference periods, assessment of these constructs in z-proso can be taken to reflect the recent tendencies for experiencing certain events/symptoms at each reporting time point. Further, the ordering of the measurement waves can provide a plausible temporal sequence to test the hypothesized relations. Finally, different reference frames can be argued to be optimal for different constructs, given differences in frequency, salience, and stability/periodicity. For example, internalizing problems might be more fleeting in nature (similar to suicidal ideation) (e.g., Lamers et al., 2018); thus, a shorter recall period can be helpful to more sensitively and accurately capture information while reducing the level of recall burden on respondents.

### Statistical Procedure

**Fig. 1:**
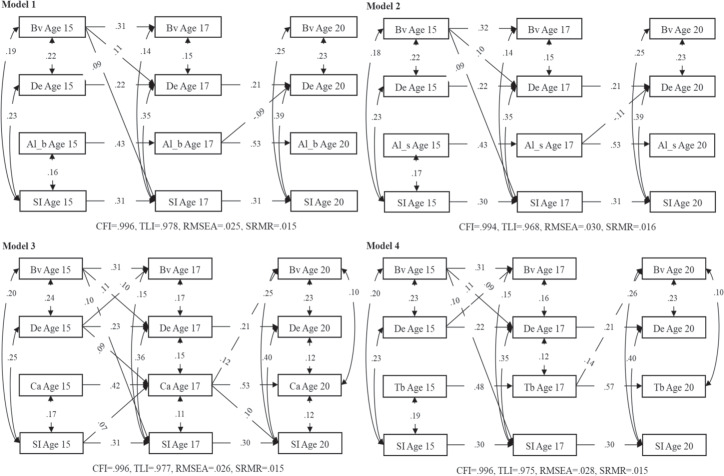
Within-person relations between bullying victimization, suicidal ideation, depressive symptoms, and substance use. SI Suicidal ideation, Bv Bullying victimization, De Depressive symptoms, Al_b Alcohol (beer/alcopops) use, Al_s Alcohol (spirits) use, Ca Cannabis use, Tb Tobacco use. Statistically significant paths are depicted only

**Fig. 2:**
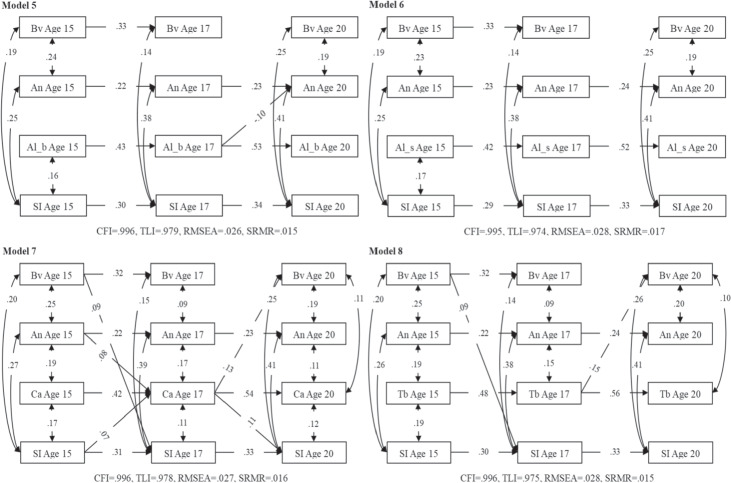
Within-person relations between bullying victimization, suicidal ideation, anxiety symptoms, and substance use. SI Suicidal ideation, Bv Bullying victimization, An Anxiety symptoms, Al_b Alcohol (beer/alcopops) use, Al_s Alcohol (spirits) use, Ca Cannabis use, Tb Tobacco use. Statistically significant paths are depicted only

Random-intercept cross-lagged panel models (RI-CLPMs; Hamaker et al., 2015) were employed to test the hypothesized longitudinal mediation models in Mplus 8.0 (Muthén & Muthén, 2015). Depressive and anxiety symptoms were examined in separate models to facilitate estimation, given their strong inter-correlation. Eight RI-CLPMs were fitted (see Figs. 1 and 2), each model including victimization, suicidal ideation, depressive (or anxiety) symptoms, and one of four forms of substance use: Tobacco, alcohol (beer/alcopops), alcohol (spirits), and cannabis. These four types of substances are viewed differently from a legal perspective in Switzerland, which also explains why they were separately examined in this study, in addition to the rationale outlined in the introduction. Specifically, cannabis use in Switzerland is illegal while the legal drinking age in Switzerland is 16 for beer, wine, and cider, and 18 for spirits, and from the age of 18, youth are permitted to purchase tobacco.

The RI-CLPM is an extension of traditional CLPM based on taking a multilevel perspective that considers that measurement occasions are nested within individuals (Hamaker et al., 2015). The RI-CLPM incorporates random intercepts to account for unobserved stable between-person variances and as such it allows the partialling out of between-person confounds (e.g., socioeconomic status) from within-person variance. This is the key difference compared to CLPM (Hamaker et al., 2015). Random intercepts were used to calculate an individual’s own over-time average levels (or typical levels) of each construct. Within-person residuals were used to account for an individual’s deviation from his or her typical level that could not explained by the between-person latent variable (i.e., random intercept).

Significant cross-lagged effects at the within-person level would indicate that, for example, individuals’ deviations from their typical levels of bullying victimization at one time point predict individuals’ fluctuations from their typical levels of suicide ideation at a later time point. Along with cross-lagged parameters, at the within-person level, RI-CLPM also includes autoregressive (which indicates the extent to which within-person deviations in a variable, e.g., suicidal ideation, are predicted by the individual’s prior deviation from the own typical levels) and within-time parameters (which indicates the extent to which within-person deviations in one variable, e.g., bullying victimization, are associated with within-person deviations in other variables, e.g., suicidal ideation, within the same measurement time point).

All models were estimated using robust maximum likelihood (MLR) estimation (treating items as continuous) for accounting for non-normality, which was deemed appropriate given that all items were on at least 5-point scales (Rhemtulla et al., 2012). Moreover, there are other advantages to treating items as continuous where it is possible. For example, diagonally weighted least squares (DWLS) employs pairwise deletion (necessitating a multiple imputation) and can also provide biased approximate fit statistics (CFI, RMSEA) (Savalei, 2020). Full information maximum likelihood estimation (FIML) was used to deal with missing data. FIML provides unbiased parameter estimates under the assumption that data are missing at random (MAR).

The significance of indirect effects was tested using maximum likelihood (ML) bootstrapping with 1000 bootstrap samples and 95% confidence intervals, as the bootstrapping procedure is unavailable for analyses based on an MLR estimator in Mplus. A bootstrapped 95% confidence interval that does not include zero can be interpreted as representing a significant indirect effect. Sex was adjusted for by regressing each intercept factor on sex.

Models were considered as of acceptable fit if a minimum cutoff of 0.90 for CFI and TLI, a maximum cutoff of 0.08 for RMSEA and SRMR (Hu & Bentler, 1999).

## Results

Means, standard deviations, and Pearson correlations between the main study variables are presented in Table S2 of Supplementary Materials.

### Within-Person Relations between Constructs

To assess bidirectional within-person relations between variables, the aforementioned eight RI-CLPM models were fitted and variables allowed to predict each other at adjacent measurement time points (i.e., from age 15 to 17 and from age 17 to 20). All the RI-CLPMs fitted the data well^1^ (see Figs. 1 and 2). Standardized autoregressive, cross-lagged, and within-time parameters of the RI-CLPMs are presented in Tables S3–S6 of Supplementary Materials and depicted in Figs. 1 and 2. The non-significant parameters (*p* > 0.050) and the covariate effects (i.e., sex) are not shown in Figs. 1 and 2 to increase clarity.

### Time-Lagged Effects

The cross-lagged parameters indicated that age 15 bullying victimization was significantly and positively associated with age 17 suicidal ideation in all tested models (*βs* = 0.09–0.10, *ps* < 0.050), with the exception of models including both anxiety symptoms and alcohol use that showed a marginally significant effect (anxiety symptoms and beer/alcopops model: *β* = 0.08, *p* = 0.051; anxiety symptoms and spirits model: *β* = 0.08, *p* = 0.055). Bullying victimization at age 15 also positively predicted depressive symptoms at age 17, with slightly different coefficient values in different substance use models (*βs* = 0.10–0.11, *ps* < 0.010). There was also a significant positive association between depressive symptoms at age 15 and bullying victimization at age 17 in models that included cannabis use (*β* = 0.10, *p* = 0.020) or tobacco use (*β* = 0.09, *p* = 0.028) but not in models that included alcohol use (beer/alcopops: *β* = 0.08, *p* = 0.062; spirits*: β* = 0.08, *p* = 0.068). And both tobacco and cannabis use at age 17 were positively related to bullying victimization at age 20, and coefficients varied slightly in models that included anxiety or depressive symptoms (*βs*_tobacco_ = 0.15/0.14, *ps* < .050; *βs*_cannabis_ = 0.13/0.12, *ps* < 0.050). Besides, age 17 cannabis use positively predicted age 20 suicidal ideation, with slightly different coefficients in the anxiety versus depressive symptoms models (*β*s = 0.11/0.10, *ps* < 0.050). There was a positive relation between age 15 suicidal ideation and age 17 cannabis use in models including depressive or anxiety symptoms (*β*s = 0.07, *ps* < 0.050). Additionally, depressive/anxiety symptoms at age 15 positively predicted cannabis use at age 17 (*β*_depression/anxiety_ = 0.09/0.08, *ps* < 0.050). However, between ages 17 and 20, alcohol (beer/alcopops) and alcohol (spirits) use negatively predicted depressive symptoms (*β*_(beer/alcopops)/(spirits)_ = −0.09/−0.11, *ps* < 0.050), as well as alcohol (beer/alcopops) use negatively predicted anxiety symptoms (*β* = −0.10, *p* = 0.021).

### Correlations within the Same Year

All within-time associations were positive and significant at ages 15, or 17, or 20, except for associations of alcohol use (spirits and beer/alcopops) with victimization/depressive/ anxiety symptoms (i.e., no significant concurrent associations between them at ages 15, 17, or 20).

### Mediation Analysis

In the next step, the mediation effects of depressive/anxiety symptoms and each form of substance use in the relation between victimization and suicidal ideation (including mediating pathways from victimization to suicidal ideation and from suicidal ideation to victimization) were tested. In all mediation models, the direct pathways (i.e., second-order cross-lagged) were also added from age 15 victimization to age 20 suicidal ideation and from age 15 suicidal ideation to age 20 victimization. Mediation analysis results showed that 95% bootstrapped confidence intervals of all indirect effects contained zero, indicating that no indirect effects were significant (see Table S7 of supplementary materials, for details). However, after adding the direct pathways, three unexpected effects were observed in the model testing mediation effects of depressive symptoms and alcohol use (beer/alcopops), i.e., from age 15 victimization to age 20 suicidal ideation (*β* = −0.23, *p* = 0.001) and from ages 15 and 17 suicidal ideation to age 20 victimization (*β*_age15/17_ = −0.56/−0.34, *p*_age15/17_ = 0.014/0.037).

### Sensitivity and Additional Analyses

Additional exploratory analyses were performed to provide more details on these associations. First, traditional CLPMs were also employed to fit the data. The results showed that age 15 bullying victimization positively predicted age 20 suicidal ideation via age 17 depressive symptoms, and the estimated mediating effects of age 17 depressive symptoms were slightly different across each substance use models (*β*s = 0.012–0.013, bootstrapped 95% confidence interval [0.004, 0.024/0.025]) (see Table S8 of supplementary materials, for details). Meanwhile, age 15 suicidal ideation was positively associated with age 20 bullying victimization through age 17 depressive symptoms, and the estimated mediating effects varied slightly in different substance use models (*β*s = 0.008–0.009, bootstrapped 95% confidence interval [0.001, 0.018/0.019]).

Second, given that previous research suggested sex/gender differences in bullying victimization (e.g., Salmon et al., 2018), substance use (e.g., Shanahan et al., 2021), internalizing problems (e.g., Murray, Ushakova et al., 2021), and suicidality (e.g., Langhinrichsen-Rohling et al., 2009), the multi-group RI-CLPM was conducted to examine potential sex differences, in which cross-lagged paths were constrained to be equal across sex in one model and freely estimated in another model. According to the Satorra–Bentler scaled chi-square difference testing (see Table S9 of supplementary materials, for details), the results showed no sex differences in the within-person cross-lagged association between bullying victimization, internalizing problems, substance use, and suicidal ideation.

Lastly, because prior studies examined how different forms of victimization associated with, for instance, suicidal ideation (e.g., Brunstein Klomek et al., 2019), the four different forms of victimization in the hypothesized mediation models were separately evaluated in the RI-CLPM, yielding a total of 32 RI-CLPM models. No mediation effects of depressive symptoms/anxiety symptoms/substance use on the within-person associations between any form of victimization and suicidal ideation were identified^2^, using 95% bootstrapped confidence intervals (see Table S10 of supplementary materials, for details).

## Discussion

A better understanding of the mechanisms underlying the association between bullying victimization and suicidal ideation among young people is important for guiding suicide interventions; however, the developmental mechanisms underlying this association have not been rigorously tested. This study investigated the potential mediating roles of depressive symptoms, anxiety symptoms, and substance use in the longitudinal relations between bullying victimization and suicidal ideation, and employed the RI-CLPM to evaluate the within-person relations between these constructs, partitioning within- and between-person sources of variance. The findings indicated that none of the depressive symptoms, anxiety symptoms, or substance use mediated the within-person relations between bullying victimization and suicidal ideation. However, this study did find a few noteworthy time-lagged within-person associations that can be used to inform interventions designed to reduce youth’s suicide risk.

### None of Mediation Effects of Internalizing Problems and Substance Use

None of hypothesized mediation effects of depressive symptoms, anxiety symptoms, and substance use on victimization-suicidality relation were confirmed, which is inconsistent with previous evidence (e.g., Duan et al., 2020). The discrepancy between the current and previous findings might be because most previous studies have employed the cross-sectional design (e.g., Duan et al., 2020) and statistical methods that did not control for potential between-person confounds (Bryson et al., 2020), whereas the current study partialled out between-person associations when estimating the links between these constructs. Indeed, the current findings using traditional CLPMs supported the mediation role of age 17 depressive symptoms in the linkage between age 15 bullying victimization and age 20 suicidal ideation and in the reverse pathway from age 15 suicidal ideation to age 20 victimization. The differences in results of RI-CLPMs and traditional CLPMs underline the importance of ruling out unmeasured between-person confounds to provide a more accurate estimate of whether and how the change in one construct is associated with the change in another construct in the temporal order (Berry & Willoughby, 2017). On the other hand, all of the mediation effects were not supported at the within-person level in the current analyses, possibly, because the time lag of the current study is relatively long (i.e., two and three years), and the relevant effects may occur in a shorter interval. Indeed, this would be consistent with the widespread within-wave associations observed in the current study. A small number of studies examining within-person associations between peer victimization and depressive symptoms with shorter assessment intervals (e.g., 6 months) indicated that the two were longitudinally and cross-sectionally associated (e.g., Davis et al., 2019), which also basically supports this notion. Future studies using brief intervals are thus needed to determine whether the mediating effects of internalizing problems and substance use in the victimization-suicidality link at the within-person level can be confirmed.

### Other Noteworthy Findings

Although no mediation effects at the within-person level were supported, there were several notable within-person findings with potential prevention implications. The results suggested that age 15 bullying victimization predicted within-person increases in age 17 suicidal ideation and depressive symptoms, which is in line with numerous previous findings (e.g., see van Geel et al., 2021 for a review). These findings go beyond most previous findings by partialling out between-person confounders, thereby supporting the notion that bullying victimization can be used as a target factor to reduce depressive symptoms and suicidal ideation at the within-person level. The finding also showed that age 15 depressive symptoms was associated with within-person increases in age 17 bullying victimization, indicating that youth experiencing higher depressive symptoms than they usually do would also be subsequently relatively more frequently exposed to bullying victimization. This is also consistent with previous findings that have indicated that characteristics associated with depressive symptoms, such as rejection sensitivity (e.g., youth with depressive symptoms display oversensitivity to victimization/social rejection), may partly explain depressive symptoms-victimization associations (e.g., Calleja et al., 2020). Future research would be beneficial to measure these perceptual and cognitive mechanisms to investigate whether they partially account for the findings here.

Additionally, the findings suggested that young people who escalated their use of tobacco or cannabis at age 17 relative to their typical level were more likely to also show relative increases in being bullied at age 20. These findings (i.e., depressive symptoms/tobacco use/cannabis use→victimization) are consistent with the stress generation model, for example, that depressed youth with significant difficulties in interpersonal problem solving or adolescent substance users (here, primarily smokers and cannabis users), potentially with increased impulsivity and aggression, may be at risk of difficult interpersonal situations such as being bullied. Further investigation is needed to examine the possible mechanisms (e.g., social problem-solving deficits, impulsivity, or aggression) underlying these links to gain insight into why depressive symptoms and tobacco and cannabis use increased the risk of bullying victimization among young people. This can then inform intervention components, for example, in anti-bullying programs. Besides this, however, the hypothesized pathways from victimization to substance use were not observed; one speculated explanation for this is that substance use is strongly socially embedded in youth. As such, those who are victimized/socially excluded may be less likely to be involved in substance use than their peers.

The findings also suggested that adolescents at age 15 experiencing higher suicidal ideation than their typical level used more cannabis at age 17, and young people at age 17 more frequently using cannabis than their typical frequency displayed greater suicidal ideation at age 20. These results were in line with the bidirectional model (e.g., Dawes et al., 2008). This suggests a possible vicious cycle whereby adolescents may use cannabis as a coping mechanism to escape or relieve suicidal feelings but its use, could reinforce vulnerable adolescents’ contemplation of suicide. Indeed, cannabis use is associated with numerous factors related to suicidal risk, such as impairments in impulse control (Rinehart & Spencer, 2021), psychosocial failure (e.g., occupational or educational failure, Marmorstein & Iacono, 2011), psychopathology (e.g., depressive symptoms, Hunt et al., 2020), or a reduction in social connections (Buckner et al., 2017) or the use of protective resources (e.g., physical activity, Ashdown-Franks et al., 2019). However, the result regarding the relation between cannabis use and depressive/anxiety symptoms only supported the self-medication hypothesis (Turner et al., 2018), suggesting cannabis might be used as a coping strategy to deal with depressive/anxiety symptoms but it does not increase the risk of anxiety and depressive symptoms.

Besides these findings, three counterintuitive results were observed in the mediation model including victimization, suicidal ideation, depressive symptoms, and alcohol use (beer/alcopops). They include a negative effect of age 15 victimization on age 20 suicidal ideation and of age 15/17 suicidal ideation on age 20 victimization. These counterintuitive results mainly involve (or are due to adding) direct pathways (i.e., second-order cross-lagged effects) and are with a long-time span (from age 15 to 20). A recent study proposed that resilience may account for detrimental effects of victimization on suicidal ideation that diminish in the long-term (Schoeler, 2018). Nonetheless, these findings are difficult to explain using existing theories, and more research is required to understand the timing and role of resilience in the relation between bullying victimization and suicidality at the within-person level.

### Implications of the Findings

Even though the mediating roles of internalizing problems and substance use in the within-person relation between victimization and suicidal ideation were not supported, the current findings, by ruling out stable between-person differences from within-person changes, could inform within-individual based intervention strategies aimed at reducing suicide risk and bullying victimization. For example, the results imply that intervention programs targeting youth experiencing increased victimization would be beneficial in reducing their suicidal thoughts and depressive symptoms; however, this is not the first study to suggest this; previous research has also indicated this (e.g., Arseneault et al., 2010). In addition to treating victimization as a target for intervention efforts, social-cognitive processes, such as critical self-referent attributions (Prinstein et al., 2005), self-blame (Perren & Ladd, 2013), and rumination (e.g., Peets & Salmivalli, 2021), have been suggested to contribute to the link between victimization and internalizing problems and can be used as intervention targets. Moreover, individuals’ increases in depressive symptoms and substance use (especially tobacco and cannabis use) should be addressed to reduce their exposure to bullying. Additionally, lowering individuals’ use of cannabis or improving their suicidal thoughts could both help break the vicious circle between cannabis use and suicidal ideation. More importantly, these within-person findings suggest the potential utility of monitoring fluctuations in these experiences, moods, or thoughts to determine periods of risk (i.e., compared to an individual’s typical level) and initiate target intervention promptly. This implication is novel in light of prior research that has mostly focused on between-person comparisons or examined these relations without controlling for between-person confounders, thus providing limited evidence for guiding change-based intervention.

### Limitations and Future Directions

The limitations of the present study should be noted. First, a single item was used to measure suicidal ideation and each form of substance use. One-item measures for assessing suicidality/substance use in the population-based samples have been widely employed in research; however, future studies using more comprehensive well-validated scales to assess these constructs are needed. The likely effect on the current study was an attenuation of some associations due to the greater measurement error that is likely to affect single-item, compared to multi-item measures. Second, this study may be limited by only using self-reports to measure bullying. This was a pragmatic design choice given that other available assessment methods, such as peer reports, may be difficult to organize, particularly in the current study with a relatively long observation period. Future studies should replicate the current findings by incorporating reports from multiple informants. Third, the longitudinal sampling intervals of the current study were relatively long; the findings are likely to inform general trends spanning a longer developmental period rather than the effects of short-term fluctuations in these constructs. Therefore, further research including data at shorter intervals or multiple scales could help get a more nuanced and precise understanding of the relations between these constructs.

Fourth, different timeframes were used to assess bullying victimization/substance use (i.e., past year) and suicidal ideation/internalizing problems (i.e., past month), which might have affected the current findings. Further studies using varying timeframes to measure these events/symptoms to explore the implications of this and to establish optimal solutions for situations where different constructs might have different optimal temporal reference frames would be helpful. Fifth, the z-proso study did not assess additional gender-related information, e.g., gender identity and sexual orientation which are known to be related to experiences of victimization. Future research should take these factors into account when examining the associations between victimization, internalizing problems, substance use, and suicidal ideation. Sixth, previous research has indicated that poly-victimization might lead to a higher risk of mental health issues than single forms of victimization (e.g., Haahr-Pedersen et al., 2020). However, given that estimating the effects of poly-victimization within RI-CLPMs is difficult, future research should go beyond examining general/single forms of victimization and examine the role of poly-victimization. Finally, measurement invariance could not be tested for the single item measures and, according to a previous study (Murray, Eisner et al., 2021), did not hold for the bullying measure.

## Conclusion

The potential mediating roles of internalizing problems and substance use in the link between bullying victimization and suicidal ideation have not been rigorously examined. This was addressed in the current study by using longitudinal data from a representative sample of Swiss youth and ruling out between-person confounds. This study found no evidence that depressive symptoms, anxiety symptoms, and substance use mediated the within-person relation between bullying victimization and suicidal ideation. However, the longitudinal within-person effects of bullying victimization on depressive symptoms and suicidal ideation were observed, and the findings also indicated that depressive symptoms, tobacco and cannabis use were key risk factors for subsequent increases in bullying victimization among young people. The results also showed that there were reciprocal reinforcing feedback loops between fluctuations in cannabis use and the same-direction changes of suicidal ideation over time. Overall, the findings suggest these variables are interrelated in a complex way over time during middle adolescence and emerging adulthood. Consideration of these relations within anti-bullying, substance use reduction, and suicide and mental health prevention programs could have the potential to improve their effectiveness with respect to not only their primary outcomes, but also additional developmentally-related outcomes.

## Supplementary Information

Supplemental Materials

## Footnotes

1 There were non-positive definite covariance matrices in the between-person level, which was mainly because the random intercept factor for suicidal ideation was strongly correlated to random intercept factors for depressive symptoms and anxiety symptoms.

2 Note, two models failed to estimate normally (no convergence).
