# Supplementary material for: Developmental Relations Between Bullying Victimization and Suicidal Ideation in Middle Adolescence and Emerging Adulthood: Do Internalizing Problems and Substance Use Mediate Their Links?
Source: J Youth Adolesc. 2022 May 14;51(9):1745–59. doi: 10.1007/s10964-022-01630-4 (PMC9279233; doi:10.1007/s10964-022-01630-4)
Supplement: Supplementary file 1 — Supplemental Materials [file 10964_2022_1630_MOESM1_ESM.docx]

**Table S1.** The numbers (percentage) of participants with valid data from at least 1, 2, or 3 time points on each construct

| **Ages 15, 17, and 20** | **Bullying victimization** | **Depressive symptoms** | **Anxiety symptoms** | **Suicidal ideation** |
| --- | --- | --- | --- | --- |
| At least 1 time points | 1465 (100.00%) | 1465 (100.00%) | 1465 (100.00%) | 1464 (99.93%) |
| At least 2 time points | 1361 (92.90%) | 1363 (93.04%) | 1363 (93.04%) | 1359 (92.76%) |
| 3 time points | 1100 (75.09%) | VV (75.22%) | VV (75.22%) | 1098 (74.95%) |
| **Ages 15, 17, and 20** | **Tobacco use** | **Alcohol(beer/alcopops) use** | **Alcohol(spirits) use** | **Cannabis use** |
| At least 1 time points | 1463 (99.86%) | 1462 (99.80%) | 1463 (99.86%) | 1463 (99.86%) |
| At least 2 time points | 1358 (92.70%) | 1355 (92.49%) | 1357 (92.63%) | 1358 (92.70%) |
| 3 time points | 1081 (73.79%) | 1069 (72.97%) | 1067 (72.83%) | 1073 (73.24%) |

**Table S2**

Descriptive statistics and Pearson correlations between study variables

|  | *n* | *M* | *SD* | 1 | 2 | 3 | 4 | 5 | 6 | 7 | 8 | 9 | 10 | 11 | 12 |
| --- | --- | --- | --- | --- | --- | --- | --- | --- | --- | --- | --- | --- | --- | --- | --- |
| 1.Age 15 Bullying victimization | 1444 | 1.64 | 0.69 | _ |  |  |  |  |  |  |  |  |  |  |  |
| 2.Age 17 Bullying victimization | 1303 | 1.45 | 0.57 | .476*** | _ |  |  |  |  |  |  |  |  |  |  |
| 3.Age 20 Bullying victimization | 1179 | 1.37 | 0.47 | .304*** | .422*** | _ |  |  |  |  |  |  |  |  |  |
| 4.Age 15 Suicidal ideation | 1439 | 1.30 | 0.71 | .203*** | .126*** | .105*** | _ |  |  |  |  |  |  |  |  |
| 5.Age 17 Suicidal ideation | 1304 | 1.37 | 0.80 | .166*** | .195*** | .174*** | .406*** | _ |  |  |  |  |  |  |  |
| 6.Age 20 Suicidal ideation | 1178 | 1.30 | 0.71 | .083** | .114*** | .282*** | .226*** | .396*** | _ |  |  |  |  |  |  |
| 7.Age 15 Depressive symptoms | 1445 | 2.37 | 0.83 | .267*** | .240*** | .177*** | .400*** | .283*** | .247*** | _ |  |  |  |  |  |
| 8.Age 17 Depressive symptoms | 1305 | 2.45 | 0.87 | .223*** | .273*** | .237*** | .283*** | .472*** | .284*** | .551*** | _ |  |  |  |  |
| 9.Age 20 Depressive symptoms | 1180 | 2.40 | 0.85 | .176*** | .208*** | .329*** | .243*** | .315*** | .499*** | .436*** | .529*** | _ |  |  |  |
| 10.Age 15 Anxiety symptoms | 1445 | 2.29 | 0.86 | .259*** | .176*** | .171*** | .411*** | .312*** | .223*** | .700*** | .474*** | .341*** | _ |  |  |
| 11.Age 17 Anxiety symptoms | 1305 | 2.38 | 0.90 | .154*** | .178*** | .188*** | .259*** | .471*** | .234*** | .460*** | .686*** | .424*** | .589*** | _ |  |
| 12.Age 20 Anxiety symptoms | 1180 | 2.40 | 0.92 | .116*** | .130*** | .269*** | .223*** | .296** | .471*** | .394*** | .480*** | .718*** | .459*** | .569*** | _ |
| 13.Age 15 Tobacco use | 1431 | 2.75 | 1.75 | 0.003 | -0.053 | 0.008 | .091** | 0.004 | -0.022 | 0.043 | 0.024 | 0.013 | .065* | 0.022 | -0.002 |
| 14.Age 17 Tobacco use | 1294 | 3.50 | 1.89 | -0.007 | -0.032 | 0.056 | .064* | 0.042 | -0.002 | .077** | .097*** | 0.048 | .087** | .103*** | 0.034 |
| 15.Age 20 Tobacco use | 1177 | 3.68 | 1.92 | -0.022 | -0.032 | .070* | 0.038 | 0.054 | 0.035 | 0.029 | .065* | 0.025 | 0.042 | .073* | 0.006 |
| 16.Age 15 Alcohol(beer/alcopops) use | 1419 | 2.27 | 1.34 | .105*** | 0.047 | .072* | .118*** | 0.031 | 0.038 | .082** | 0.050 | 0.035 | .068* | 0.029 | -0.021 |
| 17.Age 17 Alcohol((beer/alcopops) use | 1290 | 3.20 | 1.50 | .103*** | .084** | .073* | .062* | 0.041 | 0.029 | .063* | .056* | -0.004 | 0.008 | 0.027 | -.064* |
| 18.Age 20 Alcohol((beer/alcopops) use | 1177 | 3.72 | 1.39 | .073* | .067* | .104*** | 0.005 | -0.019 | 0.042 | .071* | 0.014 | 0.004 | 0.025 | -0.027 | -0.050 |
| 17.Age 15 Alcohol(spirits) use | 1426 | 2.08 | 1.27 | .053* | -0.015 | 0.042 | .116*** | 0.052 | 0.027 | .068* | .058* | 0.048 | .085** | .062* | 0.031 |
| 20.Age 17 Alcohol(spirits) use | 1284 | 2.92 | 1.40 | .088** | .066* | .060* | .065* | 0.045 | -0.003 | .092** | .104*** | 0.009 | .065* | .070* | -0.015 |
| 21.Age 20 Alcohol(spirits) use | 1177 | 3.34 | 1.25 | .060* | 0.043 | .088** | -0.014 | -0.006 | 0.011 | .064* | 0.031 | 0.015 | 0.002 | -0.022 | -.069* |
| 22.Age 15 Cannabis use | 1429 | 1.90 | 1.45 | 0.024 | -0.002 | 0.032 | .082** | -0.007 | 0.001 | 0.025 | 0.021 | -0.015 | 0.019 | -0.016 | -.065* |
| 23.Age 17 Cannabis use | 1287 | 2.47 | 1.66 | 0.042 | 0.026 | .093** | .088** | .088** | .090** | .066* | .102*** | .060* | 0.033 | .055* | 0.002 |
| 24.Age 20 Cannabis use | 1178 | 2.55 | 1.67 | 0.041 | 0.010 | .123*** | 0.041 | 0.025 | .118*** | 0.032 | .067* | .086** | -0.010 | -0.030 | 0.015 |

*Note.* **p*<0.05; ***p*<0.01; ****p*<0.001

**Table S2 (continued)**

Descriptive statistics and Pearson correlations between study variables

|  | 13 | 14 | 15 | 16 | 17 | 18 | 19 | 20 | 21 | 22 | 23 | 24 |
| --- | --- | --- | --- | --- | --- | --- | --- | --- | --- | --- | --- | --- |
| 13.Age 15 Tobacco use | _ |  |  |  |  |  |  |  |  |  |  |  |
| 14.Age 17 Tobacco use | .647*** | _ |  |  |  |  |  |  |  |  |  |  |
| 15.Age 20 Tobacco use | .492*** | .689*** | _ |  |  |  |  |  |  |  |  |  |
| 16.Age 15 Alcohol(beer/alcopops) use | .463*** | .350*** | .214*** | _ |  |  |  |  |  |  |  |  |
| 17.Age 17 Alcohol((beer/alcopops) use | .242*** | .372*** | .233*** | .563*** | _ |  |  |  |  |  |  |  |
| 18.Age 20 Alcohol((beer/alcopops) use | .194*** | .240*** | .254*** | .426*** | .628*** | _ |  |  |  |  |  |  |
| 17.Age 15 Alcohol(spirits) use | .633*** | .440*** | .330*** | .672*** | .409*** | .305*** | _ |  |  |  |  |  |
| 20.Age 17 Alcohol(spirits) use | .335*** | .487*** | .351*** | .503*** | .736*** | .530*** | .480*** | _ |  |  |  |  |
| 21.Age 20 Alcohol(spirits) use | .207*** | .293*** | .342*** | .350*** | .538*** | .775*** | .298*** | .565** | _ |  |  |  |
| 22.Age 15 Cannabis use | .602*** | .410*** | .277*** | .490*** | .353*** | .239*** | .608*** | .375** | .227*** | _ |  |  |
| 23.Age 17 Cannabis use | .425*** | .492*** | .373*** | .425*** | .505*** | .373*** | .427*** | .542** | .352*** | .571*** | _ |  |
| 24.Age 20 Cannabis use | .322*** | .389*** | .438*** | .299*** | .389*** | .374*** | .320*** | .396** | .397*** | .422*** | .630*** | _ |

*Note.* **p*<0.05; ***p*<0.01; ****p*<0.001

**Table S3**

RI-CLPM standardized parameters for bullying victimization, depressive symptoms, alcohol use, and suicidal ideation (at the within-person level)

| **Alcohol (beer/alcopops) use** | | **Estimate** | ***SE*** | ***p*** |  | **Alcohol (spirits) use** | | **Estimate** | ***SE*** | ***p*** |
| --- | --- | --- | --- | --- | --- | --- | --- | --- | --- | --- |
| Age 17 SI | ON |  |  |  |  | Age 17 SI | ON |  |  |  |
| Age 15 Bv |  | **0.09** | 0.04 | 0.034 |  | Age 15 Bv |  | **0.09** | 0.04 | 0.037 |
| Age 15 SI |  | **0.31** | 0.06 | 0.000 |  | Age 15 SI |  | **0.30** | 0.07 | 0.000 |
| Age 15 De |  | 0.01 | 0.05 | 0.922 |  | Age 15 De |  | 0.01 | 0.05 | 0.880 |
| Age 15 Al_b |  | -0.02 | 0.04 | 0.687 |  | Age 15 Al_s |  | 0.03 | 0.04 | 0.411 |
|  |  |  |  |  |  |  |  |  |  |  |
| Age 20 SI | ON |  |  |  |  | Age 20 SI | ON |  |  |  |
| Age 17 Bv |  | 0.03 | 0.05 | 0.537 |  | Age 17 Bv |  | 0.03 | 0.05 | 0.543 |
| Age 17 SI |  | **0.31** | 0.06 | 0.000 |  | Age 17 SI |  | **0.31** | 0.06 | 0.000 |
| Age 17 De |  | -0.02 | 0.05 | 0.695 |  | Age 17 De |  | -0.02 | 0.05 | 0.661 |
| Age 17 Al_b |  | 0.01 | 0.04 | 0.805 |  | Age 17 Al_s |  | -0.01 | 0.04 | 0.774 |
|  |  |  |  |  |  |  |  |  |  |  |
| Age 17 Bv | ON |  |  |  |  | Age 17 Bv | ON |  |  |  |
| Age 15 Bv |  | **0.31** | 0.05 | 0.000 |  | Age 15 Bv |  | **0.32** | 0.05 | 0.000 |
| Age 15 SI |  | 0.03 | 0.04 | 0.511 |  | Age 15 SI |  | 0.03 | 0.04 | 0.463 |
| Age 15 De |  | 0.08 | 0.04 | 0.062 |  | Age 15 De |  | 0.08 | 0.04 | 0.068 |
| Age 15 Al_b |  | -0.04 | 0.05 | 0.347 |  | Age 15 Al_s |  | -0.06 | 0.04 | 0.122 |
|  |  |  |  |  |  |  |  |  |  |  |
| Age 20 Bv | ON |  |  |  |  | Age 20 Bv | ON |  |  |  |
| Age 17 Bv |  | 0.15 | 0.09 | 0.086 |  | Age 17 Bv |  | 0.16 | 0.09 | 0.074 |
| Age 17 SI |  | 0.11 | 0.06 | 0.081 |  | Age 17 SI |  | 0.11 | 0.06 | 0.088 |
| Age 17 De |  | 0.05 | 0.06 | 0.324 |  | Age 17 De |  | 0.05 | 0.06 | 0.321 |
| Age 17 Al_b |  | 0.00 | 0.06 | 0.995 |  | Age 17 Al_s |  | 0.02 | 0.06 | 0.668 |
|  |  |  |  |  |  |  |  |  |  |  |
| Age 17 De | ON |  |  |  |  | Age 17 De | ON |  |  |  |
| Age 15 Bv |  | **0.11** | 0.04 | 0.008 |  | Age 15 Bv |  | **0.10** | 0.04 | 0.009 |
| Age 15 SI |  | 0.02 | 0.05 | 0.662 |  | Age 15 SI |  | 0.02 | 0.05 | 0.705 |
| Age 15 De |  | **0.22** | 0.06 | 0.000 |  | Age 15 De |  | **0.22** | 0.06 | 0.000 |
| Age 15 Al_b |  | -0.06 | 0.05 | 0.235 |  | Age 15 Al_s |  | -0.04 | 0.05 | 0.353 |
|  |  |  |  |  |  |  |  |  |  |  |
| Age 20 De | ON |  |  |  |  | Age 20 De | ON |  |  |  |
| Age 17 Bv |  | 0.07 | 0.05 | 0.146 |  | Age 17 Bv |  | 0.07 | 0.05 | 0.151 |
| Age 17 SI |  | 0.05 | 0.05 | 0.401 |  | Age 17 SI |  | 0.05 | 0.05 | 0.376 |
| Age 17 De |  | **0.21** | 0.06 | 0.001 |  | Age 17 De |  | **0.21** | 0.06 | 0.000 |
| Age 17 Al_b |  | **-0.09** | 0.05 | 0.043 |  | Age 17 Al_s |  | **-0.11** | 0.05 | 0.023 |
|  |  |  |  |  |  |  |  |  |  |  |
| Age 17 Al_b | ON |  |  |  |  | Age 17 Al_s | ON |  |  |  |
| Age 15 Bv |  | 0.04 | 0.03 | 0.256 |  | Age 15 Bv |  | 0.05 | 0.03 | 0.135 |
| Age 15 SI |  | 0.01 | 0.03 | 0.721 |  | Age 15 SI |  | 0.02 | 0.03 | 0.498 |
| Age 15 De |  | -0.01 | 0.04 | 0.788 |  | Age 15 De |  | 0.01 | 0.04 | 0.825 |
| Age 15 Al_b |  | **0.43** | 0.05 | 0.000 |  | Age 15 Al_s |  | **0.43** | 0.05 | 0.000 |
|  |  |  |  |  |  |  |  |  |  |  |
| Age 20 Al_b | ON |  |  |  |  | Age 20 Al_s | ON |  |  |  |
| Age 17 Bv |  | 0.01 | 0.05 | 0.811 |  | Age 17 Bv |  | 0.00 | 0.04 | 0.927 |
| Age 17 SI |  | -0.03 | 0.04 | 0.363 |  | Age 17 SI |  | 0.01 | 0.04 | 0.769 |
| Age 17 De |  | -0.05 | 0.04 | 0.202 |  | Age 17 De |  | -0.07 | 0.04 | 0.096 |
| Age 17 Al_b |  | **0.53** | 0.04 | 0.000 |  | Age 17 Al_s |  | **0.53** | 0.04 | 0.000 |
|  |  |  |  |  |  |  |  |  |  |  |
| Age 15 Bv | WITH |  |  |  |  | Age 15 Bv | WITH |  |  |  |
| Age 15 SI |  | **0.19** | 0.04 | 0.000 |  | Age 15 SI |  | **0.18** | 0.04 | 0.000 |
| Age 15 De |  | **0.22** | 0.04 | 0.000 |  | Age 15 De |  | **0.22** | 0.04 | 0.000 |
| Age 15 Al_b |  | 0.06 | 0.05 | 0.226 |  | Age 15 Al_s |  | 0.03 | 0.04 | 0.507 |
|  |  |  |  |  |  |  |  |  |  |  |
| Age 17 Bv | WITH |  |  |  |  | Age 17 Bv | WITH |  |  |  |
| Age 17 SI |  | **0.14** | 0.04 | 0.002 |  | Age 17 SI |  | **0.14** | 0.04 | 0.001 |
| Age 17 De |  | **0.15** | 0.04 | 0.000 |  | Age 17 De |  | **0.15** | 0.04 | 0.000 |
| Age 17 Al_b |  | 0.03 | 0.04 | 0.360 |  | Age 17 Al_s |  | 0.05 | 0.04 | 0.169 |
|  |  |  |  |  |  |  |  |  |  |  |
| Age 20 Bv | WITH |  |  |  |  | Age 20 Bv | WITH |  |  |  |
| Age 20 SI |  | **0.25** | 0.06 | 0.000 |  | Age 20 SI |  | **0.25** | 0.06 | 0.000 |
| Age 20 De |  | **0.23** | 0.04 | 0.000 |  | Age 20 De |  | **0.23** | 0.04 | 0.000 |
| Age 20 Al_b |  | 0.05 | 0.06 | 0.347 |  | Age 20 Al_s |  | 0.06 | 0.05 | 0.246 |
|  |  |  |  |  |  |  |  |  |  |  |
| Age 15 SI | WITH |  |  |  |  | Age 15 SI | WITH |  |  |  |
| Age 15 De |  | **0.23** | 0.06 | 0.000 |  | Age 15 De |  | **0.23** | 0.05 | 0.000 |
| Age 15 Al_b |  | **0.16** | 0.06 | 0.009 |  | Age 15 Al_s |  | **0.17** | 0.06 | 0.003 |
|  |  |  |  |  |  |  |  |  |  |  |
| Age 17 SI | WITH |  |  |  |  | Age 17 SI | WITH |  |  |  |
| Age 17 De |  | **0.35** | 0.04 | 0.000 |  | Age 17 De |  | **0.35** | 0.04 | 0.000 |
| Age 17 Al_b |  | 0.03 | 0.03 | 0.303 |  | Age 17 Al_s |  | 0.02 | 0.03 | 0.449 |
|  |  |  |  |  |  |  |  |  |  |  |
| Age 20 SI | WITH |  |  |  |  | Age 20 SI | WITH |  |  |  |
| Age 20 De |  | **0.39** | 0.04 | 0.000 |  | Age 20 De |  | **0.39** | 0.04 | 0.000 |
| Age 20 Al_b |  | 0.04 | 0.04 | 0.282 |  | Age 20 Al_s |  | 0.04 | 0.04 | 0.265 |
|  |  |  |  |  |  |  |  |  |  |  |
| Age 15 De | WITH |  |  |  |  | Age 15 De | WITH |  |  |  |
| Age 15 Al_b |  | 0.00 | 0.06 | 0.974 |  | Age 15 Al_s |  | -0.02 | 0.06 | 0.670 |
|  |  |  |  |  |  |  |  |  |  |  |
| Age 17 De | WITH |  |  |  |  | Age 17 De | WITH |  |  |  |
| Age 17 Al_b |  | 0.01 | 0.04 | 0.764 |  | Age 17 Al_s |  | 0.05 | 0.04 | 0.227 |
|  |  |  |  |  |  |  |  |  |  |  |
| Age 20 De | WITH |  |  |  |  | Age 20 De | WITH |  |  |  |
| Age 20 Al_b |  | -0.05 | 0.04 | 0.146 |  | Age 20 Al_s |  | -0.02 | 0.04 | 0.623 |

*Note.* SI= Suicidal ideation, Bv=Bullying victimization, De=Depressive symptoms, Al_b=Alcohol (beer/alcopops) use, Al_s=Alcohol (spirits) use. The values in bold represent statistically significant paths (*p*<.05).

**Table S4**

RI-CLPM standardized parameters for bullying victimization, depressive symptoms, cannabis use (or tobacco use), and suicidal ideation (at the within-person level)

| **Cannabis use** | | **Estimate** | ***SE*** | ***p*** |  | **Tobacco use** | | **Estimate** | ***SE*** | ***p*** |
| --- | --- | --- | --- | --- | --- | --- | --- | --- | --- | --- |
| Age 17 SI | ON |  |  |  |  | Age 17 SI | ON |  |  |  |
| Age 15 Bv |  | **0.10** | 0.04 | 0.026 |  | Age 15 Bv |  | **0.10** | 0.04 | 0.025 |
| Age 15 SI |  | **0.31** | 0.07 | 0.000 |  | Age 15 SI |  | **0.30** | 0.07 | 0.000 |
| Age 15 De |  | 0.02 | 0.05 | 0.674 |  | Age 15 De |  | 0.01 | 0.05 | 0.839 |
| Age 15 Ca |  | -0.01 | 0.04 | 0.865 |  | Age 15 Tb |  | 0.01 | 0.05 | 0.907 |
|  |  |  |  |  |  |  |  |  |  |  |
| Age 20 SI | ON |  |  |  |  | Age 20 SI | ON |  |  |  |
| Age 17 Bv |  | 0.04 | 0.05 | 0.336 |  | Age 17 Bv |  | 0.04 | 0.05 | 0.376 |
| Age 17 SI |  | **0.30** | 0.06 | 0.000 |  | Age 17 SI |  | **0.30** | 0.06 | 0.000 |
| Age 17 De |  | -0.02 | 0.05 | 0.711 |  | Age 17 De |  | -0.01 | 0.06 | 0.799 |
| Age 17 Ca |  | **0.10** | 0.04 | 0.024 |  | Age 17 Tb |  | 0.01 | 0.05 | 0.821 |
|  |  |  |  |  |  |  |  |  |  |  |
| Age 17 Bv | ON |  |  |  |  | Age 17 Bv | ON |  |  |  |
| Age 15 Bv |  | **0.31** | 0.05 | 0.000 |  | Age 15 Bv |  | **0.31** | 0.05 | 0.000 |
| Age 15 SI |  | 0.04 | 0.04 | 0.392 |  | Age 15 SI |  | 0.04 | 0.04 | 0.358 |
| Age 15 De |  | **0.10** | 0.04 | 0.020 |  | Age 15 De |  | **0.09** | 0.04 | 0.028 |
| Age 15 Ca |  | -0.03 | 0.04 | 0.483 |  | Age 15 Tb |  | -0.05 | 0.05 | 0.334 |
|  |  |  |  |  |  |  |  |  |  |  |
| Age 20 Bv | ON |  |  |  |  | Age 20 Bv | ON |  |  |  |
| Age 17 Bv |  | 0.16 | 0.09 | 0.074 |  | Age 17 Bv |  | 0.16 | 0.09 | 0.074 |
| Age 17 SI |  | 0.11 | 0.06 | 0.072 |  | Age 17 SI |  | 0.11 | 0.06 | 0.065 |
| Age 17 De |  | 0.05 | 0.06 | 0.373 |  | Age 17 De |  | 0.04 | 0.06 | 0.431 |
| Age 17 Ca |  | **0.12** | 0.05 | 0.023 |  | Age 17 Tb |  | **0.14** | 0.06 | 0.013 |
|  |  |  |  |  |  |  |  |  |  |  |
| Age 17 De | ON |  |  |  |  | Age 17 De | ON |  |  |  |
| Age 15 Bv |  | **0.11** | 0.04 | 0.004 |  | Age 15 Bv |  | **0.11** | 0.04 | 0.006 |
| Age 15 SI |  | 0.02 | 0.05 | 0.704 |  | Age 15 SI |  | 0.01 | 0.05 | 0.831 |
| Age 15 De |  | **0.23** | 0.06 | 0.000 |  | Age 15 De |  | **0.22** | 0.06 | 0.000 |
| Age 15 Ca |  | 0.06 | 0.05 | 0.223 |  | Age 15 Tb |  | 0.03 | 0.06 | 0.666 |
|  |  |  |  |  |  |  |  |  |  |  |
| Age 20 De | ON |  |  |  |  | Age 20 De | ON |  |  |  |
| Age 17 Bv |  | 0.08 | 0.05 | 0.099 |  | Age 17 Bv |  | 0.07 | 0.05 | 0.127 |
| Age 17 SI |  | 0.05 | 0.05 | 0.384 |  | Age 17 SI |  | 0.04 | 0.05 | 0.405 |
| Age 17 De |  | **0.21** | 0.06 | 0.001 |  | Age 17 De |  | **0.21** | 0.06 | 0.000 |
| Age 17 Ca |  | 0.07 | 0.05 | 0.112 |  | Age 17 Tb |  | 0.01 | 0.05 | 0.911 |
|  |  |  |  |  |  |  |  |  |  |  |
| Age 17 Ca | ON |  |  |  |  | Age 17 Tb | ON |  |  |  |
| Age 15 Bv |  | 0.00 | 0.03 | 0.963 |  | Age 15 Bv |  | -0.01 | 0.03 | 0.846 |
| Age 15 SI |  | **0.07** | 0.04 | 0.042 |  | Age 15 SI |  | 0.04 | 0.04 | 0.270 |
| Age 15 De |  | **0.09** | 0.04 | 0.034 |  | Age 15 De |  | 0.06 | 0.04 | 0.205 |
| Age 15 Ca |  | **0.42** | 0.06 | 0.000 |  | Age 15 Tb |  | **0.48** | 0.06 | 0.000 |
|  |  |  |  |  |  |  |  |  |  |  |
| Age 20 Ca | ON |  |  |  |  | Age 20 Tb | ON |  |  |  |
| Age 17 Bv |  | -0.01 | 0.03 | 0.890 |  | Age 17 Bv |  | -0.01 | 0.04 | 0.871 |
| Age 17 SI |  | -0.02 | 0.03 | 0.510 |  | Age 17 SI |  | 0.05 | 0.04 | 0.143 |
| Age 17 De |  | 0.07 | 0.04 | 0.064 |  | Age 17 De |  | -0.02 | 0.04 | 0.651 |
| Age 17 Ca |  | **0.53** | 0.05 | 0.000 |  | Age 17 Tb |  | **0.57** | 0.05 | 0.000 |
|  |  |  |  |  |  |  |  |  |  |  |
| Age 15 Bv | WITH |  |  |  |  | Age 15 Bv | WITH |  |  |  |
| Age 15 SI |  | **0.20** | 0.04 | 0.000 |  | Age 15 SI |  | **0.20** | 0.04 | 0.000 |
| Age 15 De |  | **0.24** | 0.04 | 0.000 |  | Age 15 De |  | **0.23** | 0.04 | 0.000 |
| Age 15 Ca |  | 0.03 | 0.05 | 0.580 |  | Age 15 Tb |  | 0.06 | 0.05 | 0.240 |
|  |  |  |  |  |  |  |  |  |  |  |
| Age 17 Bv | WITH |  |  |  |  | Age 17 Bv | WITH |  |  |  |
| Age 17 SI |  | **0.15** | 0.04 | 0.001 |  | Age 17 SI |  | **0.15** | 0.04 | 0.001 |
| Age 17 De |  | **0.17** | 0.04 | 0.000 |  | Age 17 De |  | **0.16** | 0.04 | 0.000 |
| Age 17 Ca |  | 0.02 | 0.04 | 0.584 |  | Age 17 Tb |  | 0.02 | 0.04 | 0.647 |
|  |  |  |  |  |  |  |  |  |  |  |
| Age 20 Bv | WITH |  |  |  |  | Age 20 Bv | WITH |  |  |  |
| Age 20 SI |  | **0.25** | 0.06 | 0.000 |  | Age 20 SI |  | **0.26** | 0.06 | 0.000 |
| Age 20 De |  | **0.23** | 0.04 | 0.000 |  | Age 20 De |  | **0.23** | 0.04 | 0.000 |
| Age 20 Ca |  | **0.10** | 0.05 | 0.022 |  | Age 20 Tb |  | **0.10** | 0.04 | 0.018 |
|  |  |  |  |  |  |  |  |  |  |  |
| Age 15 SI | WITH |  |  |  |  | Age 15 SI | WITH |  |  |  |
| Age 15 De |  | **0.25** | 0.05 | 0.000 |  | Age 15 De |  | **0.23** | 0.05 | 0.000 |
| Age 15 Ca |  | **0.17** | 0.06 | 0.005 |  | Age 15 Tb |  | **0.19** | 0.06 | 0.003 |
|  |  |  |  |  |  |  |  |  |  |  |
| Age 17 SI | WITH |  |  |  |  | Age 17 SI | WITH |  |  |  |
| Age 17 De |  | **0.36** | 0.04 | 0.000 |  | Age 17 De |  | **0.35** | 0.04 | 0.000 |
| Age 17 Ca |  | **0.11** | 0.03 | 0.001 |  | Age 17 Tb |  | 0.06 | 0.04 | 0.100 |
|  |  |  |  |  |  |  |  |  |  |  |
| Age 20 SI | WITH |  |  |  |  | Age 20 SI | WITH |  |  |  |
| Age 20 De |  | **0.40** | 0.04 | 0.000 |  | Age 20 De |  | **0.40** | 0.04 | 0.000 |
| Age 20 Ca |  | **0.12** | 0.04 | 0.002 |  | Age 20 Tb |  | 0.06 | 0.04 | 0.079 |
|  |  |  |  |  |  |  |  |  |  |  |
| Age 15 De | WITH |  |  |  |  | Age 15 De | WITH |  |  |  |
| Age 15 Ca |  | 0.12 | 0.06 | 0.060 |  | Age 15 Tb |  | 0.10 | 0.07 | 0.135 |
|  |  |  |  |  |  |  |  |  |  |  |
| Age 17 De | WITH |  |  |  |  | Age 17 De | WITH |  |  |  |
| Age 17 Ca |  | **0.15** | 0.04 | 0.000 |  | Age 17 Tb |  | **0.12** | 0.04 | 0.003 |
|  |  |  |  |  |  |  |  |  |  |  |
| Age 20 De | WITH |  |  |  |  | Age 20 De | WITH |  |  |  |
| Age 20 Ca |  | **0.12** | 0.04 | 0.001 |  | Age 20 Tb |  | 0.01 | 0.04 | 0.706 |

*Note.* SI= Suicidal ideation, Bv=Bullying victimization, De=Depressive symptoms, Ca=Cannabis use, Tb=Tobacco use. The values in bold represent statistically significant paths (*p*<.05).

**Table S5**

RI-CLPM standardized parameters for bullying victimization, anxiety symptoms, alcohol use, and suicidal ideation (at the within-person level)

| **Alcohol (beer/alcopops) use** | | **Estimate** | ***SE*** | ***p*** |  | **Alcohol (spirits)use** | | **Estimate** | ***SE*** | ***p*** |
| --- | --- | --- | --- | --- | --- | --- | --- | --- | --- | --- |
| Age 17 SI | ON |  |  |  |  | Age 17 SI | ON |  |  |  |
| Age 15 Bv |  | 0.08 | 0.04 | 0.051 |  | Age 15 Bv |  | 0.08 | 0.04 | 0.055 |
| Age 15 SI |  | **0.30** | 0.07 | 0.000 |  | Age 15 SI |  | **0.29** | 0.07 | 0.000 |
| Age 15 An |  | 0.05 | 0.05 | 0.317 |  | Age 15 An |  | 0.05 | 0.05 | 0.266 |
| Age 15 Al_b |  | -0.02 | 0.04 | 0.658 |  | Age 15 Al_s |  | 0.03 | 0.04 | 0.429 |
|  |  |  |  |  |  |  |  |  |  |  |
| Age 20 SI | ON |  |  |  |  | Age 20 SI | ON |  |  |  |
| Age 17 Bv |  | 0.02 | 0.04 | 0.604 |  | Age 17 Bv |  | 0.02 | 0.04 | 0.612 |
| Age 17 SI |  | **0.34** | 0.06 | 0.000 |  | Age 17 SI |  | **0.33** | 0.06 | 0.000 |
| Age 17 An |  | -0.08 | 0.05 | 0.113 |  | Age 17 An |  | -0.07 | 0.05 | 0.148 |
| Age 17 Al_b |  | 0.01 | 0.04 | 0.753 |  | Age 17 Al_s |  | -0.01 | 0.04 | 0.861 |
|  |  |  |  |  |  |  |  |  |  |  |
| Age 17 Bv | ON |  |  |  |  | Age 17 Bv | ON |  |  |  |
| Age 15 Bv |  | **0.33** | 0.05 | 0.000 |  | Age 15 Bv |  | **0.33** | 0.05 | 0.000 |
| Age 15 SI |  | 0.04 | 0.04 | 0.284 |  | Age 15 SI |  | 0.05 | 0.04 | 0.245 |
| Age 15 An |  | -0.02 | 0.04 | 0.700 |  | Age 15 An |  | -0.01 | 0.04 | 0.735 |
| Age 15 Al_b |  | -0.04 | 0.05 | 0.422 |  | Age 15 Al_s |  | -0.06 | 0.04 | 0.126 |
|  |  |  |  |  |  |  |  |  |  |  |
| Age 20 Bv | ON |  |  |  |  | Age 20 Bv | ON |  |  |  |
| Age 17 Bv |  | 0.14 | 0.09 | 0.113 |  | Age 17 Bv |  | 0.15 | 0.09 | 0.099 |
| Age 17 SI |  | 0.12 | 0.07 | 0.069 |  | Age 17 SI |  | 0.12 | 0.07 | 0.071 |
| Age 17 An |  | 0.02 | 0.06 | 0.773 |  | Age 17 An |  | 0.01 | 0.06 | 0.810 |
| Age 17 Al_b |  | 0.01 | 0.06 | 0.862 |  | Age 17 Al_s |  | 0.03 | 0.06 | 0.542 |
|  |  |  |  |  |  |  |  |  |  |  |
| Age 17 An | ON |  |  |  |  | Age 17 An | ON |  |  |  |
| Age 15 Bv |  | 0.04 | 0.04 | 0.334 |  | Age 15 Bv |  | 0.04 | 0.04 | 0.390 |
| Age 15 SI |  | -0.03 | 0.05 | 0.548 |  | Age 15 SI |  | -0.03 | 0.05 | 0.562 |
| Age 15 An |  | **0.22** | 0.07 | 0.001 |  | Age 15 An |  | **0.23** | 0.07 | 0.001 |
| Age 15 Al_b |  | 0.01 | 0.05 | 0.876 |  | Age 15 Al_s |  | 0.03 | 0.05 | 0.479 |
|  |  |  |  |  |  |  |  |  |  |  |
| Age 20 An | ON |  |  |  |  | Age 20 An | ON |  |  |  |
| Age 17 Bv |  | 0.01 | 0.05 | 0.919 |  | Age 17 Bv |  | 0.00 | 0.05 | 0.985 |
| Age 17 SI |  | 0.03 | 0.05 | 0.539 |  | Age 17 SI |  | 0.04 | 0.05 | 0.483 |
| Age 17 An |  | **0.23** | 0.06 | 0.000 |  | Age 17 An |  | **0.24** | 0.06 | 0.000 |
| Age 17 Al_b |  | **-0.10** | 0.04 | 0.021 |  | Age 17 Al_s |  | -0.07 | 0.04 | 0.092 |
|  |  |  |  |  |  |  |  |  |  |  |
| Age 17 Al_b | ON |  |  |  |  | Age 17 Al_s | ON |  |  |  |
| Age 15 Bv |  | 0.05 | 0.03 | 0.117 |  | Age 15 Bv |  | 0.06 | 0.03 | 0.077 |
| Age 15 SI |  | 0.02 | 0.03 | 0.592 |  | Age 15 SI |  | 0.03 | 0.03 | 0.365 |
| Age 15 An |  | -0.05 | 0.04 | 0.233 |  | Age 15 An |  | -0.01 | 0.04 | 0.895 |
| Age 15 Al_b |  | **0.43** | 0.05 | 0.000 |  | Age 15 Al_s |  | **0.42** | 0.05 | 0.000 |
|  |  |  |  |  |  |  |  |  |  |  |
| Age 20 Al_b | ON |  |  |  |  | Age 20 Al_s | ON |  |  |  |
| Age 17 Bv |  | 0.01 | 0.05 | 0.894 |  | Age 17 Bv |  | 0.00 | 0.04 | 0.999 |
| Age 17 SI |  | -0.03 | 0.04 | 0.416 |  | Age 17 SI |  | 0.02 | 0.04 | 0.628 |
| Age 17 An |  | -0.05 | 0.04 | 0.245 |  | Age 17 An |  | -0.07 | 0.04 | 0.115 |
| Age 17 Al_b |  | **0.53** | 0.04 | 0.000 |  | Age 17 Al_s |  | **0.52** | 0.04 | 0.000 |
|  |  |  |  |  |  |  |  |  |  |  |
| Age 15 Bv | WITH |  |  |  |  | Age 15 Bv | WITH |  |  |  |
| Age 15 SI |  | **0.19** | 0.04 | 0.000 |  | Age 15 SI |  | **0.19** | 0.04 | 0.000 |
| Age 15 An |  | **0.24** | 0.04 | 0.000 |  | Age 15 An |  | **0.23** | 0.04 | 0.000 |
| Age 15 Al_b |  | 0.07 | 0.05 | 0.129 |  | Age 15 Al_s |  | 0.04 | 0.04 | 0.362 |
|  |  |  |  |  |  |  |  |  |  |  |
| Age 17 Bv | WITH |  |  |  |  | Age 17 Bv | WITH |  |  |  |
| Age 17 SI |  | **0.14** | 0.04 | 0.002 |  | Age 17 SI |  | **0.14** | 0.04 | 0.001 |
| Age 17 An |  | 0.07 | 0.05 | 0.096 |  | Age 17 An |  | 0.08 | 0.04 | 0.087 |
| Age 17 Al_b |  | 0.04 | 0.04 | 0.355 |  | Age 17 Al_s |  | 0.06 | 0.04 | 0.142 |
|  |  |  |  |  |  |  |  |  |  |  |
| Age 20 Bv | WITH |  |  |  |  | Age 20 Bv | WITH |  |  |  |
| Age 20 SI |  | **0.25** | 0.06 | 0.000 |  | Age 20 SI |  | **0.25** | 0.06 | 0.000 |
| Age 20 An |  | **0.19** | 0.04 | 0.000 |  | Age 20 An |  | **0.19** | 0.04 | 0.000 |
| Age 20 Al_b |  | 0.05 | 0.06 | 0.335 |  | Age 20 Al_s |  | 0.07 | 0.05 | 0.214 |
|  |  |  |  |  |  |  |  |  |  |  |
| Age 15 SI | WITH |  |  |  |  | Age 15 SI | WITH |  |  |  |
| Age 15 An |  | **0.25** | 0.06 | 0.000 |  | Age 15 An |  | **0.25** | 0.06 | 0.000 |
| Age 15 Al_b |  | **0.16** | 0.06 | 0.008 |  | Age 15 Al_s |  | **0.17** | 0.06 | 0.002 |
|  |  |  |  |  |  |  |  |  |  |  |
| Age 17 SI | WITH |  |  |  |  | Age 17 SI | WITH |  |  |  |
| Age 17 An |  | **0.38** | 0.04 | 0.000 |  | Age 17 An |  | **0.38** | 0.04 | 0.000 |
| Age 17 Al_b |  | 0.03 | 0.03 | 0.268 |  | Age 17 Al_s |  | 0.03 | 0.03 | 0.407 |
|  |  |  |  |  |  |  |  |  |  |  |
| Age 20 SI | WITH |  |  |  |  | Age 20 SI | WITH |  |  |  |
| Age 20 An |  | **0.41** | 0.04 | 0.000 |  | Age 20 An |  | **0.41** | 0.04 | 0.000 |
| Age 20 Al_b |  | 0.04 | 0.04 | 0.306 |  | Age 20 Al_s |  | 0.05 | 0.04 | 0.235 |
|  |  |  |  |  |  |  |  |  |  |  |
| Age 15 An | WITH |  |  |  |  | Age 15 An | WITH |  |  |  |
| Age 15 Al_b |  | 0.07 | 0.06 | 0.251 |  | Age 15 Al_s |  | 0.08 | 0.06 | 0.157 |
|  |  |  |  |  |  |  |  |  |  |  |
| Age 17 An | WITH |  |  |  |  | Age 17 An | WITH |  |  |  |
| Age 17 Al_b |  | 0.04 | 0.04 | 0.316 |  | Age 17 Al_s |  | 0.05 | 0.04 | 0.207 |
|  |  |  |  |  |  |  |  |  |  |  |
| Age 20 An | WITH |  |  |  |  | Age 20 An | WITH |  |  |  |
| Age 20 Al_b |  | -0.04 | 0.04 | 0.362 |  | Age 20 Al_s |  | -0.06 | 0.04 | 0.100 |

*Note.* SI= Suicidal ideation, Bv=Bullying victimization, An=Anxiety symptoms, Al_b=Alcohol (beer/alcopops) use, Al_s=Alcohol (spirits) use. The values in bold represent statistically significant paths (*p*<.05).

**Table S6**

RI-CLPM standardized parameters for bullying victimization, anxiety symptoms, cannabis use (or tobacco use), and suicidal ideation (at the within-person level)

| **Cannabis use** | | **Estimate** | ***SE*** | ***p*** |  | **Tobacco use** | | **Estimate** | ***SE*** | ***p*** |
| --- | --- | --- | --- | --- | --- | --- | --- | --- | --- | --- |
| Age 17 SI | ON |  |  |  |  | Age 17 SI | ON |  |  |  |
| Age 15 Bv |  | **0.09** | 0.04 | 0.042 |  | Age 15 Bv |  | **0.09** | 0.04 | 0.040 |
| Age 15 SI |  | **0.31** | 0.07 | 0.000 |  | Age 15 SI |  | **0.30** | 0.07 | 0.000 |
| Age 15 An |  | 0.06 | 0.05 | 0.196 |  | Age 15 An |  | 0.05 | 0.05 | 0.288 |
| Age 15 Ca |  | -0.01 | 0.04 | 0.761 |  | Age 15 Tb |  | 0.00 | 0.05 | 0.953 |
|  |  |  |  |  |  |  |  |  |  |  |
| Age 20 SI | ON |  |  |  |  | Age 20 SI | ON |  |  |  |
| Age 17 Bv |  | 0.04 | 0.04 | 0.379 |  | Age 17 Bv |  | 0.04 | 0.05 | 0.413 |
| Age 17 SI |  | **0.33** | 0.06 | 0.000 |  | Age 17 SI |  | **0.33** | 0.06 | 0.000 |
| Age 17 An |  | -0.08 | 0.05 | 0.099 |  | Age 17 An |  | -0.07 | 0.05 | 0.145 |
| Age 17 Ca |  | **0.11** | 0.04 | 0.011 |  | Age 17 Tb |  | 0.02 | 0.05 | 0.704 |
|  |  |  |  |  |  |  |  |  |  |  |
| Age 17 Bv | ON |  |  |  |  | Age 17 Bv | ON |  |  |  |
| Age 15 Bv |  | **0.32** | 0.05 | 0.000 |  | Age 15 Bv |  | **0.32** | 0.05 | 0.000 |
| Age 15 SI |  | 0.05 | 0.04 | 0.200 |  | Age 15 SI |  | 0.05 | 0.04 | 0.211 |
| Age 15 An |  | 0.00 | 0.04 | 0.970 |  | Age 15 An |  | 0.01 | 0.04 | 0.913 |
| Age 15 Ca |  | -0.02 | 0.05 | 0.633 |  | Age 15 Tb |  | -0.04 | 0.05 | 0.444 |
|  |  |  |  |  |  |  |  |  |  |  |
| Age 20 Bv | ON |  |  |  |  | Age 20 Bv | ON |  |  |  |
| Age 17 Bv |  | 0.15 | 0.09 | 0.095 |  | Age 17 Bv |  | 0.15 | 0.09 | 0.096 |
| Age 17 SI |  | 0.12 | 0.07 | 0.062 |  | Age 17 SI |  | 0.12 | 0.07 | 0.059 |
| Age 17 An |  | 0.01 | 0.06 | 0.866 |  | Age 17 An |  | 0.01 | 0.06 | 0.870 |
| Age 17 Ca |  | **0.13** | 0.05 | 0.018 |  | Age 17 Tb |  | **0.15** | 0.06 | 0.010 |
|  |  |  |  |  |  |  |  |  |  |  |
| Age 17 An | ON |  |  |  |  | Age 17 An | ON |  |  |  |
| Age 15 Bv |  | 0.05 | 0.04 | 0.198 |  | Age 15 Bv |  | 0.05 | 0.04 | 0.220 |
| Age 15 SI |  | -0.03 | 0.05 | 0.598 |  | Age 15 SI |  | -0.04 | 0.05 | 0.470 |
| Age 15 An |  | **0.22** | 0.07 | 0.001 |  | Age 15 An |  | **0.22** | 0.07 | 0.002 |
| Age 15 Ca |  | 0.11 | 0.06 | 0.062 |  | Age 15 Tb |  | 0.08 | 0.06 | 0.225 |
|  |  |  |  |  |  |  |  |  |  |  |
| Age 20 An | ON |  |  |  |  | Age 20 An | ON |  |  |  |
| Age 17 Bv |  | 0.01 | 0.05 | 0.768 |  | Age 17 Bv |  | 0.02 | 0.05 | 0.758 |
| Age 17 SI |  | 0.03 | 0.05 | 0.529 |  | Age 17 SI |  | 0.03 | 0.05 | 0.594 |
| Age 17 An |  | **0.23** | 0.06 | 0.000 |  | Age 17 An |  | **0.24** | 0.06 | 0.000 |
| Age 17 Ca |  | 0.06 | 0.04 | 0.152 |  | Age 17 Tb |  | 0.00 | 0.05 | 0.937 |
|  |  |  |  |  |  |  |  |  |  |  |
| Age 17 Ca | ON |  |  |  |  | Age 17 Tb | ON |  |  |  |
| Age 15 Bv |  | 0.00 | 0.03 | 0.978 |  | Age 15 Bv |  | -0.01 | 0.03 | 0.805 |
| Age 15 SI |  | **0.07** | 0.04 | 0.039 |  | Age 15 SI |  | 0.03 | 0.04 | 0.335 |
| Age 15 An |  | **0.08** | 0.04 | 0.046 |  | Age 15 An |  | 0.07 | 0.04 | 0.132 |
| Age 15 Ca |  | **0.42** | 0.07 | 0.000 |  | Age 15 Tb |  | **0.48** | 0.06 | 0.000 |
|  |  |  |  |  |  |  |  |  |  |  |
| Age 20 Ca | ON |  |  |  |  | Age 20 Tb | ON |  |  |  |
| Age 17 Bv |  | 0.01 | 0.03 | 0.879 |  | Age 17 Bv |  | -0.01 | 0.04 | 0.829 |
| Age 17 SI |  | 0.01 | 0.04 | 0.837 |  | Age 17 SI |  | 0.04 | 0.04 | 0.297 |
| Age 17 An |  | -0.02 | 0.04 | 0.711 |  | Age 17 An |  | 0.02 | 0.04 | 0.676 |
| Age 17 Ca |  | **0.54** | 0.05 | 0.000 |  | Age 17 Tb |  | **0.56** | 0.05 | 0.000 |
|  |  |  |  |  |  |  |  |  |  |  |
| Age 15 Bv | WITH |  |  |  |  | Age 15 Bv | WITH |  |  |  |
| Age 15 SI |  | **0.20** | 0.04 | 0.000 |  | Age 15 SI |  | **0.20** | 0.04 | 0.000 |
| Age 15 An |  | **0.25** | 0.04 | 0.000 |  | Age 15 An |  | **0.25** | 0.04 | 0.000 |
| Age 15 Ca |  | 0.03 | 0.05 | 0.585 |  | Age 15 Tb |  | 0.06 | 0.05 | 0.207 |
|  |  |  |  |  |  |  |  |  |  |  |
| Age 17 Bv | WITH |  |  |  |  | Age 17 Bv | WITH |  |  |  |
| Age 17 SI |  | **0.15** | 0.04 | 0.001 |  | Age 17 SI |  | **0.14** | 0.04 | 0.001 |
| Age 17 An |  | **0.09** | 0.04 | 0.043 |  | Age 17 An |  | **0.09** | 0.05 | 0.044 |
| Age 17 Ca |  | 0.03 | 0.04 | 0.447 |  | Age 17 Tb |  | 0.02 | 0.04 | 0.538 |
|  |  |  |  |  |  |  |  |  |  |  |
| Age 20 Bv | WITH |  |  |  |  | Age 20 Bv | WITH |  |  |  |
| Age 20 SI |  | **0.25** | 0.06 | 0.000 |  | Age 20 SI |  | **0.26** | 0.06 | 0.000 |
| Age 20 An |  | **0.19** | 0.04 | 0.000 |  | Age 20 An |  | **0.20** | 0.04 | 0.000 |
| Age 20 Ca |  | **0.11** | 0.05 | 0.021 |  | Age 20 Tb |  | **0.10** | 0.04 | 0.018 |
|  |  |  |  |  |  |  |  |  |  |  |
| Age 15 SI | WITH |  |  |  |  | Age 15 SI | WITH |  |  |  |
| Age 15 An |  | **0.27** | 0.06 | 0.000 |  | Age 15 An |  | **0.26** | 0.06 | 0.000 |
| Age 15 Ca |  | **0.17** | 0.06 | 0.005 |  | Age 15 Tb |  | **0.19** | 0.06 | 0.003 |
|  |  |  |  |  |  |  |  |  |  |  |
| Age 17 SI | WITH |  |  |  |  | Age 17 SI | WITH |  |  |  |
| Age 17 An |  | **0.39** | 0.04 | 0.000 |  | Age 17 An |  | **0.38** | 0.04 | 0.000 |
| Age 17 Ca |  | **0.11** | 0.03 | 0.001 |  | Age 17 Tb |  | 0.05 | 0.04 | 0.121 |
|  |  |  |  |  |  |  |  |  |  |  |
| Age 20 SI | WITH |  |  |  |  | Age 20 SI | WITH |  |  |  |
| Age 20 An |  | **0.41** | 0.04 | 0.000 |  | Age 20 An |  | **0.41** | 0.04 | 0.000 |
| Age 20 Ca |  | **0.12** | 0.04 | 0.003 |  | Age 20 Tb |  | 0.06 | 0.04 | 0.083 |
|  |  |  |  |  |  |  |  |  |  |  |
| Age 15 An | WITH |  |  |  |  | Age 15 An | WITH |  |  |  |
| Age 15 Ca |  | **0.19** | 0.06 | 0.003 |  | Age 15 Tb |  | **0.19** | 0.07 | 0.006 |
|  |  |  |  |  |  |  |  |  |  |  |
| Age 17 An | WITH |  |  |  |  | Age 17 An | WITH |  |  |  |
| Age 17 Ca |  | **0.17** | 0.04 | 0.000 |  | Age 17 Tb |  | **0.15** | 0.04 | 0.000 |
|  |  |  |  |  |  |  |  |  |  |  |
| Age 20 An | WITH |  |  |  |  | Age 20 An | WITH |  |  |  |
| Age 20 Ca |  | **0.11** | 0.04 | 0.003 |  | Age 20 Tb |  | 0.01 | 0.04 | 0.891 |

*Note.* SI= Suicidal ideation, Bv=Bullying victimization, An=Anxiety symptoms, Ca=Cannabis use, Tb=Tobacco use. The values in bold represent statistically significant paths (*p*<.05).

**Table S7**

**RI-CLPM standardized indirect effects**

| **Mediation paths** | **Estimated** | **95% CI** |
| --- | --- | --- |
| **In the model including bullying victimization, depressive symptoms, alcohol (beer/alcopops) use, and suicidal ideation** | | |
| Age 15 Bv → Age17 De → Age 20 SI | 0.000 | [-0.019, 0.011] |
| Age 15 Bv → Age17 Be → Age 20 SI | 0.001 | [-0.004, 0.006] |
| Age 15 SI → Age17 De → Age 20 Bv | 0.002 | [-0.008, 0.018] |
| Age 15 SI → Age17 Be → Age 20 Bv | 0.000 | [-0.006, 0.006] |
| **In the model including bullying victimization, depressive symptoms, alcohol (spirits) use, and suicidal ideation** | | |
| Age 15 Bv → Age17 De → Age 20 SI | 0.000 | [-0.012, 0.010] |
| Age 15 Bv → Age17 Sp → Age 20 SI | 0.001 | [-0.004, 0.005] |
| Age 15 SI → Age17 De → Age 20 Bv | 0.004 | [-0.009, 0.022] |
| Age 15 SI → Age17 Sp → Age 20 Bv | 0.001 | [-0.009, 0.013] |
| **In the model including bullying victimization, depressive symptoms, cannabis use, and suicidal ideation** | | |
| Age 15 Bv → Age17 De → Age 20 SI | -0.001 | [-0.023, 0.010] |
| Age 15 Bv → Age17 Ca → Age 20 SI | 0.001 | [-0.008, 0.009] |
| Age 15 SI → Age17 De → Age 20 Bv | 0.001 | [-0.008, 0.013] |
| Age 15 SI → Age17 Ca → Age 20 Bv | 0.011 | [-0.003, 0.034] |
| **In the model including bullying victimization, depressive symptoms, tobacco use, and suicidal ideation** | | |
| Age 15 Bv → Age17 De → Age 20 SI | -0.001 | [-0.023, 0.010] |
| Age 15 Bv → Age17 Tb → Age 20 SI | 0.000 | [-0.005, 0.003] |
| Age 15 SI → Age17 De → Age 20 Bv | 0.001 | [-0.007, 0.010] |
| Age 15 SI → Age17 Tb → Age 20 Bv | 0.005 | [-0.005,0.018] |
| **In the model including bullying victimization, anxiety symptoms, alcohol (beer/alcopops) use, and suicidal ideation** | | |
| Age 15 Bv → Age17 An → Age 20 SI | -0.003 | [-0.017, 0.009] |
| Age 15 Bv → Age17 Be → Age 20 SI | 0.001 | [-0.005, 0.006] |
| Age 15 SI → Age17 An → Age 20 Bv | -0.001 | [-0.010, 0.006] |
| Age 15 SI → Age17 Be → Age 20 Bv | 0.000 | [-0.005, 0.007] |
| **In the model including bullying victimization, anxiety symptoms, alcohol (spirits) use, and suicidal ideation** | | |
| Age 15 Bv → Age17 An → Age 20 SI | 0.002 | [-0.012, 0.012] |
| Age 15 Bv → Age17 Sp → Age 20 SI | 0.001 | [-0.006, 0.007] |
| Age 15 SI → Age17 An → Age 20 Bv | -0.001 | [-0.011, 0.006] |
| Age 15 SI → Age17 Sp → Age 20 Bv | 0.002 | [-0.007, 0.016] |
| **In the model including bullying victimization, anxiety symptoms, cannabis use, and suicidal ideation** | | |
| Age 15 Bv → Age17 An → Age 20 SI | -0.005 | [-0.022, 0.007] |
| Age 15 Bv → Age17 Ca → Age 20 SI | 0.000 | [-0.010, 0.010] |
| Age 15 SI → Age17 An → Age 20 Bv | 0.000 | [-0.009, 0.006] |
| Age 15 SI → Age17 Ca → Age 20 Bv | 0.008 | [-0.002, 0.035] |
| **In the model including bullying victimization, anxiety symptoms, tobacco use, and suicidal ideation** | | |
| Age 15 Bv → Age17 An → Age 20 SI | -0.005 | [-0.022, 0.007] |
| Age 15 Bv → Age17 Tb → Age 20 SI | 0.000 | [-0.005, 0.003] |
| Age 15 SI → Age17 An → Age 20 Bv | 0.000 | [-0.008, 0.005] |
| Age 15 SI → Age17 Tb → Age 20 Bv | 0.004 | [-0.005, 0.016] |

*Note.* SI= Suicidal ideation, Bv=Bullying victimization, De=Depressive symptoms, An=Anxiety symptoms, Al_b=Alcohol (beer/alcopops) use, Al_s=Alcohol (spirits) use, Ca=Cannabis use, Tb=Tobacco use.

**Table S8**

**CLPM standardized indirect effects**

| **Mediation paths** | **Estimated** | **95% CI** |
| --- | --- | --- |
| **In the model including bullying victimization, depressive symptoms, alcohol (beer/alcopops) use, and suicidal ideation** | | |
| Age 15 Bv → Age17 De → Age 20 SI | **0.012** | [0.005, 0.025] |
| Age 15 Bv → Age17 Be → Age 20 SI | 0.000 | [-0.002, 0.003] |
| Age 15 SI → Age17 De → Age 20 Bv | **0.009** | [0.001, 0.019] |
| Age 15 SI → Age17 Be → Age 20 Bv | -0.001 | [-0.004, 0.001] |
| **In the model including bullying victimization, depressive symptoms, alcohol (spirits) use, and suicidal ideation** | | |
| Age 15 Bv → Age17 De → Age 20 SI | **0.013** | [0.005, 0.025] |
| Age 15 Bv → Age17 Sp → Age 20 SI | -0.001 | [-0.006, 0.001] |
| Age 15 SI → Age17 De → Age 20 Bv | **0.009** | [0.001, 0.019] |
| Age 15 SI → Age17 Sp → Age 20 Bv | 0.000 | [-0.003, 0.001] |
| **In the model including bullying victimization, depressive symptoms, cannabis use, and suicidal ideation** | | |
| Age 15 Bv → Age17 De → Age 20 SI | **0.012** | [0.004, 0.024] |
| Age 15 Bv → Age17 Ca → Age 20 SI | 0.000 | [-0.003, 0.003] |
| Age 15 SI → Age17 De → Age 20 Bv | **0.008** | [0.001, 0.018] |
| Age 15 SI → Age17 Ca → Age 20 Bv | 0.003 | [-0.001, 0.009] |
| **In the model including bullying victimization, depressive symptoms, tobacco use, and suicidal ideation** | | |
| Age 15 Bv → Age17 De → Age 20 SI | **0.013** | [0.005, 0.025] |
| Age 15 Bv → Age17 Tb → Age 20 SI | 0.000 | [-0.001, 0.003] |
| Age 15 SI → Age17 De → Age 20 Bv | **0.008** | [0.001, 0.018] |
| Age 15 SI → Age17 Tb → Age 20 Bv | 0.000 | [-0.003, 0.002] |
| **In the model including bullying victimization, anxiety symptoms, alcohol (beer/alcopops) use, and suicidal ideation** | | |
| Age 15 Bv → Age17 An → Age 20 SI | 0.004 | [0.000, 0.010] |
| Age 15 Bv → Age17 Be → Age 20 SI | 0.001 | [-0.002, 0.004] |
| Age 15 SI → Age17 An → Age 20 Bv | 0.004 | [-0.005, 0.014] |
| Age 15 SI → Age17 Be → Age 20 Bv | 0.000 | [-0.002, 0.002] |
| **In the model including bullying victimization, anxiety symptoms, alcohol (spirits) use, and suicidal ideation** | | |
| Age 15 Bv → Age17 An → Age 20 SI | 0.004 | [0.000, 0.010] |
| Age 15 Bv → Age17 Sp → Age 20 SI | -0.001 | [-0.006, 0.002] |
| Age 15 SI → Age17 An → Age 20 Bv | 0.004 | [-0.005, 0.013] |
| Age 15 SI → Age17 Sp → Age 20 Bv | 0.000 | [-0.002, 0.002] |
| **In the model including bullying victimization, anxiety symptoms, cannabis use, and suicidal ideation** | | |
| Age 15 Bv → Age17 An → Age 20 SI | 0.003 | [0.000, 0.009] |
| Age 15 Bv → Age17 Ca → Age 20 SI | 0.000 | [-0.002, 0.004] |
| Age 15 SI → Age17 An → Age 20 Bv | 0.004 | [-0.005, 0.013] |
| Age 15 SI → Age17 Ca → Age 20 Bv | 0.003 | [0.000, 0.009] |
| **In the model including bullying victimization, anxiety symptoms, tobacco use, and suicidal ideation** | | |
| Age 15 Bv → Age17 An → Age 20 SI | 0.004 | [0.000, 0.010] |
| Age 15 Bv → Age17 Tb → Age 20 SI | 0.000 | [-0.001, 0.003] |
| Age 15 SI → Age17 An → Age 20 Bv | 0.004 | [-0.004, 0.013] |
| Age 15 SI → Age17 Tb → Age 20 Bv | 0.000 | [-0.003, 0.002] |

*Note.* SI= Suicidal ideation, Bv=Bullying victimization, De=Depressive symptoms, An=Anxiety symptoms, Al_b=Alcohol (beer/alcopops) use, Al_s=Alcohol (spirits) use, Ca=Cannabis use, Tb=Tobacco use. The values in bold represent statistically significant indirect effects (i.e., 95% confidence interval that does not include zero).

**Table S9**

**Sex differences testing in the within-person association among bullying victimization, internalizing problems, substance use, and suicidal ideation**

| **Models** | Δ*S-Bχ*^2^ (24) | *p* |
| --- | --- | --- |
| **In the model including bullying victimization, depressive symptoms, alcohol (beer/alcopops) use, and suicidal ideation** | | |
|  | 23.513 | 0.490 |
| **In the model including bullying victimization, depressive symptoms, alcohol (spirits) use, and suicidal ideation** | | |
|  | 28.699 | 0.232 |
| **In the model including bullying victimization, depressive symptoms, cannabis use, and suicidal ideation** | | |
|  | 28.487 | 0.240 |
| **In the model including bullying victimization, depressive symptoms, tobacco use, and suicidal ideation** | | |
|  | 35.646 | 0.059 |
| **In the model including bullying victimization, anxiety symptoms, alcohol (beer/alcopops) use, and suicidal ideation** | | |
|  | 20.387 | 0.675 |
| **In the model including bullying victimization, anxiety symptoms, alcohol (spirits) use, and suicidal ideation** | | |
|  | 29.061 | 0.218 |
| **In the model including bullying victimization, anxiety symptoms, cannabis use, and suicidal ideation** | | |
|  | 24.152 | 0.453 |
| **In the model including bullying victimization, anxiety symptoms, tobacco use, and suicidal ideation** | | |
|  | 28.970 | 0.221 |

**Table S10**

**RI-CLPM standardized indirect effects concerning each form of bullying victimization**

| **Mediation paths** | **Estimated** | **95% CI** |
| --- | --- | --- |
| **In the model including bullying victimization, depressive symptoms, alcohol (beer/alcopops) use, and suicidal ideation** | | |
| Age 15 SV → Age17 De → Age 20 SI | 0.000 | [-0.012, 0.005] |
| Age 15 SV → Age17 Be → Age 20 SI | 0.000 | [-0.003, 0.005] |
| Age 15 SI → Age17 De → Age 20 SV | 0.002 | [-0.007, 0.018] |
| Age 15 SI → Age17 Be → Age 20 SV | 0.000 | [-0.005, 0.007] |
|  |  |  |
| Age 15 VV → Age17 De → Age 20 SI | 0.000 | [-0.020, 0.013] |
| Age 15 VV → Age17 Be → Age 20 SI | 0.001 | [-0.003, 0.007] |
| Age 15 SI → Age17 De → Age 20 VV | 0.002 | [-0.006, 0.017] |
| Age 15 SI → Age17 Be → Age 20 VV | 0.001 | [-0.003, 0.008] |
|  |  |  |
| Age 15 PV → Age17 De → Age 20 SI | -0.001 | [-0.009, 0.005] |
| Age 15 PV → Age17 Be → Age 20 SI | 0.000 | [-0.002, 0.003] |
| Age 15 SI → Age17 De → Age 20 PV | -0.001 | [-0.016, 0.009] |
| Age 15 SI → Age17 Be → Age 20 PV | -0.001 | [-0.010, 0.005] |
|  |  |  |
| Age 15 PDV → Age17 De → Age 20 SI | -0.001 | [-0.011, 0.006] |
| Age 15 PDV → Age17 Be → Age 20 SI | 0.000 | [-0.003, 0.004] |
| Age 15 SI → Age17 De → Age 20 PDV | 0.003 | [-0.008, 0.017] |
| Age 15 SI → Age17 Be → Age 20 PDV | -0.001 | [-0.007, 0.006] |
| **In the model including bullying victimization, depressive symptoms, alcohol (spirits) use, and suicidal ideation** | | |
| Age 15 SV → Age17 De → Age 20 SI | 0.000 | [-0.007, 0.006] |
| Age 15 SV → Age17 Sp → Age 20 SI | 0.000 | [-0.003, 0.005] |
| Age 15 SI → Age17 De → Age 20 SV | 0.003 | [-0.008, 0.020] |
| Age 15 SI → Age17 Sp → Age 20 SV | 0.000 | [-0.008, 0.008] |
|  |  |  |
| Age 15 VV → Age17 De → Age 20 SI | 0.001 | [-0.016, 0.013] |
| Age 15 VV→ Age17 Sp → Age 20 SI | 0.000 | [-0.004, 0.004] |
| Age 15 SI → Age17 De → Age 20 VV | 0.004 | [-0.006, 0.018] |
| Age 15 SI → Age17 Sp → Age 20 VV | 0.002 | [-0.005, 0.011] |
|  |  |  |
| Age 15 PV → Age17 De → Age 20 SI | -0.001 | [-0.008, 0.004] |
| Age 15 PV→ Age17 Sp → Age 20 SI | -0.001 | [-0.008, 0.005] |
| Age 15 SI → Age17 De → Age 20 PV | -0.001 | [-0.014, 0.010] |
| Age 15 SI → Age17 Sp → Age 20 PV | 0.000 | [-0.008, 0.006] |
|  |  |  |
| Age 15 PDV → Age17 De → Age 20 SI | -0.001 | [-0.011, 0.005] |
| Age 15 PDV → Age17 Sp → Age 20 SI | 0.000 | [-0.005, 0.004] |
| Age 15 SI → Age17 De → Age 20 PDV | 0.002 | [-0.008, 0.017] |
| Age 15 SI → Age17 Sp → Age 20 PDV | -0.003 | [-0.011, 0.007] |
| **In the model including bullying victimization, depressive symptoms, cannabis use, and suicidal ideation** | | |
| Age 15 SV → Age17 De → Age 20 SI | -0.002 | [-0.017, 0.005] |
| Age 15 SV → Age17 Ca → Age 20 SI | 0.000 | [-0.010, 0.010] |
| Age 15 SI → Age17 De → Age 20 SV | 0.000 | [-0.008, 0.009] |
| Age 15 SI → Age17 Ca → Age 20 SV | 0.007 | [-0.003, 0.026] |
|  |  |  |
| Age 15 VV → Age17 De → Age 20 SI | -0.001 | [-0.024, 0.013] |
| Age 15 VV → Age17 Ca → Age 20 SI | 0.001 | [-0.007, 0.009] |
| Age 15 SI → Age17 De → Age 20 VV | 0.001 | [-0.007, 0.014] |
| Age 15 SI → Age17 Ca → Age 20 VV | 0.007 | [-0.004, 0.026] |
|  |  |  |
| Age 15 PV → Age17 De → Age 20 SI | NO CONVERGENCE | |
| Age 15 PV → Age17 Ca → Age 20 SI |  |  |
| Age 15 SI → Age17 De → Age 20 PV |  |  |
| Age 15 SI → Age17 Ca → Age 20 PV |  |  |
|  |  |  |
| Age 15 PDV → Age17 De → Age 20 SI | -0.001 | [-0.012, 0.006] |
| Age 15 PDV → Age17 Ca → Age 20 SI | -0.002 | [-0.009, 0.006] |
| Age 15 SI → Age17 De → Age 20 PDV | 0.002 | [-0.009, 0.016] |
| Age 15 SI → Age17 Ca → Age 20 PDV | 0.007 | [-0.007, 0.027] |
| **In the model including bullying victimization, depressive symptoms, tobacco use, and suicidal ideation** | | |
| Age 15 SV → Age17 De → Age 20 SI | -0.002 | [-0.014, 0.006] |
| Age 15 SV→ Age17 Tb → Age 20 SI | 0.000 | [-0.005, 0.005] |
| Age 15 SI → Age17 De → Age 20 SV | 0.000 | [-0.008, 0.009] |
| Age 15 SI → Age17 Tb → Age 20 SV | 0.004 | [-0.006,0.016] |
|  |  |  |
| Age 15 VV → Age17 De → Age 20 SI | 0.001 | [-0.023, 0.013] |
| Age 15 VV→ Age17 Tb → Age 20 SI | 0.000 | [-0.005, 0.005] |
| Age 15 SI → Age17 De → Age 20 VV | 0.001 | [-0.006, 0.011] |
| Age 15 SI → Age17 Tb → Age 20 VV | 0.003 | [-0.003,0.011] |
|  |  |  |
| Age 15 PV → Age17 De → Age 20 SI | NO CONVERGENCE | |
| Age 15 PV→ Age17 Tb → Age 20 SI |  |  |
| Age 15 SI → Age17 De → Age 20 PV |  |  |
| Age 15 SI → Age17 Tb → Age 20 PV |  |  |
|  |  |  |
| Age 15 PDV → Age17 De → Age 20 SI | -0.001 | [-0.013, 0.006] |
| Age 15 PDV → Age17 Tb → Age 20 SI | 0.000 | [-0.005, 0.004] |
| Age 15 SI → Age17 De → Age 20 PDV | 0.001 | [-0.010, 0.013] |
| Age 15 SI → Age17 Tb → Age 20 PDV | 0.002 | [-0.003,0.013] |
| **In the model including bullying victimization, anxiety symptoms, alcohol (beer/alcopops) use, and suicidal ideation** | | |
| Age 15 SV → Age17 An → Age 20 SI | -0.001 | [-0.018, 0.011] |
| Age 15 SV → Age17 Be → Age 20 SI | 0.001 | [-0.003, 0.005] |
| Age 15 SI → Age17 An → Age 20 SV | 0.000 | [-0.008, 0.007] |
| Age 15 SI → Age17 Be → Age 20 SV | 0.000 | [-0.005, 0.007] |
|  |  |  |
| Age 15 VV → Age17 An → Age 20 SI | -0.008 | [-0.029, 0.004] |
| Age 15 VV → Age17 Be → Age 20 SI | 0.001 | [-0.005, 0.007] |
| Age 15 SI → Age17 An → Age 20 VV | -0.002 | [-0.012, 0.006] |
| Age 15 SI → Age17 Be → Age 20 VV | 0.001 | [-0.003, 0.009] |
|  |  |  |
| Age 15 PV → Age17 An → Age 20 SI | -0.001 | [-0.008, 0.007] |
| Age 15 PV → Age17 Be → Age 20 SI | 0.000 | [-0.002, 0.003] |
| Age 15 SI → Age17 An → Age 20 PV | -0.001 | [-0.009, 0.007] |
| Age 15 SI → Age17 Be → Age 20 PV | -0.002 | [-0.011, 0.004] |
|  |  |  |
| Age 15 PDV → Age17 An → Age 20 SI | 0.004 | [-0.007, 0.018] |
| Age 15 PDV → Age17 Be → Age 20 SI | 0.001 | [-0.003, 0.005] |
| Age 15 SI → Age17 An → Age 20 PDV | 0.000 | [-0.007, 0.007] |
| Age 15 SI → Age17 Be → Age 20 PDV | -0.001 | [-0.006, 0.006] |
| **In the model including bullying victimization, anxiety symptoms, alcohol (spirits) use, and suicidal ideation** | | |
| Age 15 SV → Age17 An → Age 20 SI | 0.003 | [-0.013, 0.015] |
| Age 15 SV → Age17 Sp → Age 20 SI | 0.000 | [-0.004, 0.004] |
| Age 15 SI → Age17 An → Age 20 SV | 0.000 | [-0.009, 0.008] |
| Age 15 SI → Age17 Sp → Age 20 SV | 0.001 | [-0.006, 0.011] |
|  |  |  |
| Age 15 VV → Age17 An → Age 20 SI | -0.006 | [-0.024, 0.004] |
| Age 15 VV → Age17 Sp → Age 20 SI | 0.000 | [-0.005, 0.005] |
| Age 15 SI → Age17 An → Age 20 VV | -0.002 | [-0.012, 0.005] |
| Age 15 SI → Age17 Sp → Age 20 VV | 0.002 | [-0.003, 0.013] |
|  |  |  |
| Age 15 PV → Age17 An → Age 20 SI | 0.000 | [-0.007, 0.009] |
| Age 15 PV → Age17 Sp → Age 20 SI | -0.001 | [-0.007, 0.005] |
| Age 15 SI → Age17 An → Age 20 PV | -0.000 | [-0.009, 0.007] |
| Age 15 SI → Age17 Sp → Age 20 PV | 0.000 | [-0.006, 0.007] |
|  |  |  |
| Age 15 PDV→ Age17 An → Age 20 SI | 0.005 | [-0.005, 0.020] |
| Age 15 PDV→ Age17 Sp → Age 20 SI | 0.000 | [-0.005, 0.005] |
| Age 15 SI → Age17 An → Age 20 PDV | -0.001 | [-0.008, 0.008] |
| Age 15 SI → Age17 Sp → Age 20 PDV | -0.002 | [-0.010, 0.008] |
| **In the model including bullying victimization, anxiety symptoms, cannabis use, and suicidal ideation** | | |
| Age 15 SV → Age17 An → Age 20 SI | -0.008 | [-0.028, 0.004] |
| Age 15 SV → Age17 Ca → Age 20 SI | 0.000 | [-0.011, 0.011] |
| Age 15 SI → Age17 An → Age 20 SV | 0.001 | [-0.009, 0.007] |
| Age 15 SI → Age17 Ca → Age 20 SV | 0.007 | [-0.003, 0.025] |
|  |  |  |
| Age 15 VV → Age17 An → Age 20 SI | -0.011 | [-0.035, 0.004] |
| Age 15 VV → Age17 Ca → Age 20 SI | 0.000 | [-0.009, 0.009] |
| Age 15 SI → Age17 An → Age 20 VV | -0.001 | [-0.011, 0.005] |
| Age 15 SI → Age17 Ca → Age 20 VV | 0.004 | [-0.004, 0.023] |
|  |  |  |
| Age 15 PV → Age17 An → Age 20 SI | 0.004 | [-0.007, 0.014] |
| Age 15 PV → Age17 Ca → Age 20 SI | 0.002 | [-0.005, 0.011] |
| Age 15 SI → Age17 An → Age 20 PV | 0.000 | [-0.010, 0.007] |
| Age 15 SI → Age17 Ca → Age 20 PV | 0.005 | [-0.010, 0.019] |
|  |  |  |
| Age 15 PDV → Age17 An → Age 20 SI | 0.004 | [-0.008, 0.018] |
| Age 15 PDV → Age17 Ca → Age 20 SI | -0.002 | [-0.011, 0.006] |
| Age 15 SI → Age17 An → Age 20 PDV | 0.000 | [-0.007, 0.009] |
| Age 15 SI → Age17 Ca → Age 20 PDV | 0.009 | [-0.008, 0.027] |
| **In the model including bullying victimization, anxiety symptoms, tobacco use, and suicidal ideation** | | |
| Age 15 SV → Age17 An → Age 20 SI | -0.007 | [-0.027, 0.006] |
| Age 15 SV → Age17 Tb → Age 20 SI | 0.000 | [-0.005, 0.006] |
| Age 15 SI → Age17 An → Age 20 SV | 0.001 | [-0.008, 0.009] |
| Age 15 SI → Age17 Tb → Age 20 SV | 0.003 | [-0.006, 0.014] |
|  |  |  |
| Age 15 VV→ Age17 An → Age 20 SI | -0.010 | [-0.034, 0.004] |
| Age 15 VV → Age17 Tb → Age 20 SI | 0.000 | [-0.005, 0.003] |
| Age 15 SI → Age17 An → Age 20 VV | -0.001 | [-0.011, 0.005] |
| Age 15 SI → Age17 Tb → Age 20 VV | 0.002 | [-0.003, 0.010] |
|  |  |  |
| Age 15 PV→ Age17 An → Age 20 SI | -0.002 | [-0.007, 0.012] |
| Age 15 PV → Age17 Tb → Age 20 SI | 0.000 | [-0.004, 0.003] |
| Age 15 SI → Age17 An → Age 20 PV | -0.001 | [-0.011, 0.007] |
| Age 15 SI → Age17 Tb → Age 20 PV | 0.004 | [-0.005, 0.019] |
|  |  |  |
| Age 15 PDV → Age17 An → Age 20 SI | 0.001 | [-0.008, 0.015] |
| Age 15 PDV → Age17 Tb → Age 20 SI | -0.001 | [-0.006, 0.004] |
| Age 15 SI → Age17 An → Age 20 PDV | 0.000 | [-0.007, 0.008] |
| Age 15 SI → Age17 Tb → Age 20 PDV | 0.002 | [-0.004, 0.012] |

*Note.* SI= Suicidal ideation, Bv=Bullying victimization, SV=Social victimization; VV=Verbal victimization; PV=Physical victimization; PDV=Property damage victimization, De=Depressive symptoms, An=Anxiety symptoms, Al_b=Alcohol (beer/alcopops) use, Al_s=Alcohol (spirits) use, Ca=Cannabis use, Tb=Tobacco use.
